# Assesment and interpretation of negative forelimb allometry in the evolution of non-avian Theropoda

**DOI:** 10.1186/s12983-019-0342-9

**Published:** 2019-12-02

**Authors:** José A. Palma Liberona, Sergio Soto-Acuña, Marco A. Mendez, Alexander O. Vargas

**Affiliations:** 1 Laboratorio de Ontogenia y Filogenia, Departamento de Biología, Facultad de Ciencias, Universidad de Chile., Las Palmeras 3425, Santiago, Chile; 2 Laboratorio de Genética y Evolución, Departamento de Ciencias Ecológicas, Facultad de Ciencias, Universidad de Chile., Las Palmeras 3425, Santiago, Chile

**Keywords:** Theropoda, Allometry, Evolution, Phylogeny, Ontogeny, Paedomorphosis

## Abstract

**Background:**

The origin of birds is marked by a significant decrease in body size along with an increase in relative forelimb size. However, before the evolution of flight, both traits may have already been related: It has been proposed that an evolutionary trend of negative forelimb allometry existed in non-avian Theropoda, such that larger species often have relatively shorter forelimbs. Nevertheless, several exceptions exist, calling for rigorous phylogenetic statistical testing.

**Results:**

Here, we re-assessed allometric patterns in the evolution of non-avian theropods, for the first time taking into account the non-independence among related species due to shared evolutionary history.

We confirmed a main evolutionary trend of negative forelimb allometry for non-avian Theropoda, but also found support that some specific subclades (Coelophysoidea, Ornithomimosauria, and Oviraptorosauria) exhibit allometric trends that are closer to isometry, losing the ancestral negative forelimb allometry present in Theropoda as a whole.

**Conclusions:**

Explanations for negative forelimb allometry in the evolution of non-avian theropods have not been discussed, yet evolutionary allometric trends often reflect ontogenetic allometries, which suggests negative allometry of the forelimb in the ontogeny of most non-avian theropods. In modern birds, allometric growth of the limbs is related to locomotor and behavioral changes along ontogeny. After reviewing the evidence for such changes during the ontogeny of non-avian dinosaurs, we propose that proportionally longer arms of juveniles became adult traits in the small-sized and paedomorphic Aves.

## Background

Along the theropod-bird transition, many functional and morphological transformations took place, including a potential for flight, which is the focal point of several studies (e.g. references [1–5] among others). Two key morphological changes required for flight were the reduction of body size [6, 7] and the increase in relative forelimb size [8, 9]. However, both of these changes had already begun in non-avian Theropoda, long before the origin of avian flight. Further, they were likely related to each other: An evolutionary trend of negative allometry appears to have existed in non-avian theropods, such that species with a larger body size often have proportionally smaller forelimbs [10, 11]. Nevertheless, it is clear this was not always the case: some large-sized species have proportionally larger arms, while other small-sized species have proportionally smaller arms. Based on such observations, a lack of negative allometry has been suggested within Therizinosauria [11] and Ornithomimosauria [10]. Additionally, Aves evolved an opposite trend of positive allometry (proportionally larger arms in larger species) that is already present among Mesozoic taxa [11], and continues in modern Neornithes [12]. The evidence for a diversity of evolutionary trends opens the question of whether the data indeed supports the existence of a main trend of negative forelimb allometry for non-avian theropods. This question should be approached through phylogenetic statistical methods, that properly consider the non-independence of data from related specimens when estimating allometric coefficients. It has been argued that incorrect branch lengths can have an effect on Phylogenetic Independent Contrasts, therefore raw regression values have been used and then compared with ancestral node reconstruction values to argue that phylogeny did not overly affect estimated parameters [11]. However, without correcting or taking into account the covariation of non-independent data, regressions may incur in bias for the estimated parameters as well as elevated type I errors [13].

Here, we have re-assessed the evidence for evolutionary allometric trends of the forelimb in non-avian theropods, using the largest dataset to date, new combinations of statistical tools, and an assessment of how specific subclades within Theropoda. may have evolved different evolutionary trends. Our results confirm negative allometry as the main trend for non-avian Theropoda, and allow us to formally identify those theropod subclades that likely had a different trend. To explain evolutionary allometric trends, we discuss how these are related to the allometric growth of the limbs in the ontogeny of modern birds, and these in turn to changes in behavior and locomotion along development. We review the evidence for forelimb function and allometry in the ontogeny of non-avian dinosaurs, and the potential relation of body size reduction, paedomorphosis, and forelimb size near the origin of birds.

## Materials and methods

### Measurements

We collected measurements of 163 fossil specimens within Theropoda across 108 genera (Additional file 2), including species represented by multiple specimens. We aimed to identify the allometric trends that existed previous to flight specialization and positive allometric trends of Aves; therefore, no avian taxa were included, considering Aves as the clade of volant theropods containing the last common ancestor of *Archaeopteryx lithographica* and *Vultur gryphus,* and all its descendants [14]. This approach was conservative in that we still included several fossil taxa that might have been volant, and that are placed within Aves by some authors, namely *Anchiornis huxleyi*, *Aurornis xui* (proposed as a synonym of *Anchiornis* in [15]), *Xiaotingia zhengi*, *Eosinopteryx brevipenna* and *Serikornis sungei*. These taxa were labelled as “*Anchiornis*-related taxa” in this study. We also included Scansoriopterygidae, which have been placed within Aves by some authors [7], but also in a non-avian position (within Avialae in [16]; and as the sister clade of Oviraptorosauria in [17]). Likewise, we did not exclude Troodontidae or Dromeosauridae, despite containing potentially volant forms, and despite proposed phylogenetic positions that would place them within Aves as defined above [18, 19].

Measurements of femoral and humeral length (FL and HL respectively) were collected from original published descriptions and studies confirming that humerus and femur were well preserved and non-controversial. This likely introduced some variation due to possible methodological differences in the way measurements were taken by different authors, but also allowed for a dataset of worldwide specimens that is much larger than could be afforded given the restrictions in time and resources that are required for taking all measurements in person. FL was selected given that it is a good proxy for body size [7, 20–22] and is conserved and reported more often than other measurements such as snout-vent length. Therefore, even though FL can be subject to specific evolution (decoupling from body size variation [11]), it allowed us to maximize the number of specimens in our dataset. We chose HL over other forelimb elements since the humerus is most often conserved and reported, even though other elements may potentially show stronger negative allometry [23]. Following standard procedure, all collected measurements were log10 transformed allowing us to fit our data using linear models instead of power law equations. The complete dataset can be found in the supporting online information (Additional file 2).

Our dataset included all specimens, regardless of ontogenetic stage, and regardless of whether some species were represented by multiple individuals. One concern that arises is whether species with multiple individuals may be over-represented and generate bias. Although phylogenetic methods can arguably address this problem [24], we also tested this question by producing a second reduced dataset, with only one specimen per species, which was selected as the single largest specimen of that species. Another possible concern is that adults and juveniles should not be considered together in the same analysis, in order to properly distinguish evolutionary trends from ontogenetic trends (regardless of whether they may be related). To test if mixed ontogenetic status produced any significant differences, we generated a dataset comprised only by adults, in order to observe “pure” evolutionary trends. We considered a juvenile to be a young animal with no signs of impending maturity that would place it as an adult or sub-adult (following [25]).This “adult” dataset was maximally conservative, in that we excluded every specimen that has been proposed to be a juvenile, without independent assessment of ontogenetic status or standing controversies (which are common [25];). We also generated a dataset composed only of specimens proposed to be juveniles, to allow a preliminary examination of any evidence that juveniles may have had a different allometric trend.

### Phylogenetic relationships

**Fig. 1:**
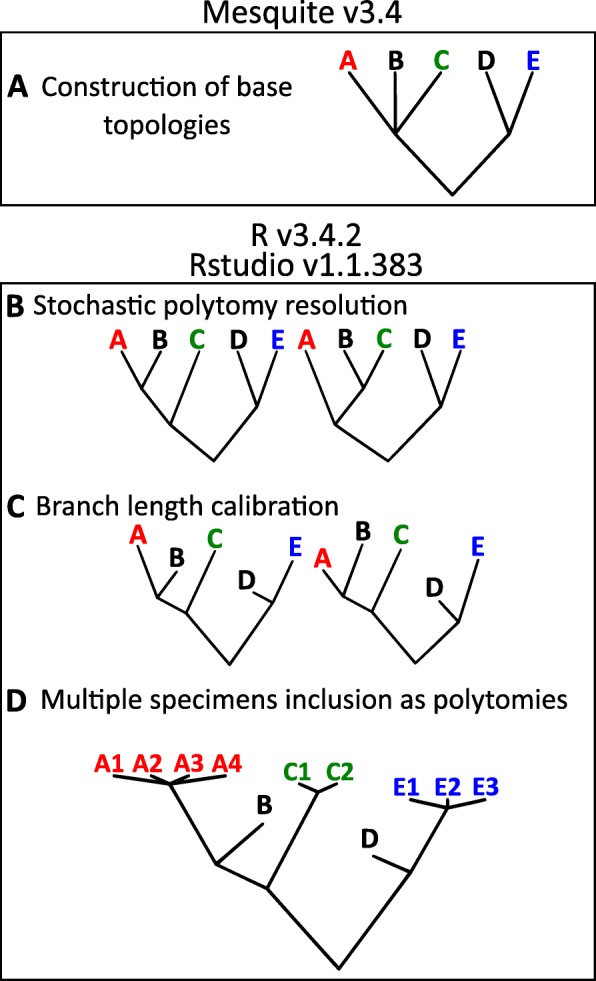
Tree construction and calibration workflow. **a** base topologies are manually constructed in Mesquite, **b** base topologies are exported to RStudio and their polytomies stochastically resolved, **c** Branch lengths are calibrated using species age, 1000 time calibrated trees are generated for each topology in steps (**b)** and (**c**, **d**) branch tips of species represented by multiple specimens are replaced with a branch length 0 polytomy in order to account for intraspecific variation

Although the main phylogenetic relationships within Theropoda are mostly agreed upon, some aspects are still under discussion, leading to phylogenetic uncertainties (unsolved polytomies) that have an effect on parameter estimation [26–29]. For this study, we created 6 topologies as informal supertrees. The polytomies present on these topologies were then stochastically resolved and their branch lengths calibrated using a stochastic sampling method (Fig. 1) generating 1000 trees per topology with variations in topology and branch lengths.

The six supertrees used were constructed using Mesquite v3.4 [30] (Fig. 1a). We labeled these topologies with numbers 1 through 6, these are: 1) considering Megaraptora closer to *Allosaurus* than to *Tyrannosaurus* [31], with Troodontidae and Scansoriopterygidae closer to Aves than Dromaeosauridae, while *Anchiornis* and related taxa (*Xiaotingia*, *Aurornis*, *Eosinopteryx* and *Serikornis*) are closer to Aves than to Troodontidae [7]; 2) similar to topology 1 but considering a clade Megaraptora closer to *Tyrannosaurus* than to *Allosaurus* [32, 33]; 3) placing Megaraptora closer to *Allosaurus* than to *Tyrannosaurus*, Dromaeosauridae and Troodontidae closer to each other than either is to Aves, and Scansoriopterygidae as the sister group to Aves [16]; 4) similar to topology 3 but with Megaraptora closer to *Tyrannosaurus* than to *Allosaurus*; 5) considering Megaraptora as closer to *Allosaurus* than to *Tyrannosaurus*; Dromaeosauridae closer to Aves than Troodontidae and Scansoriopterygidae as the sister taxa of Oviraptorosauria [17]; and 6) similar to topology 5 but placing Megaraptora closer to *Tyrannosaurus* than to *Allosaurus*. In order to maximize the species represented in the topologies we used a similar method to that described in [29] inserting those species absent from published phylogenies in the most resolved non-controversial polytomy. The constructed supertrees are shown in the supporting information (Additional file 1: Figure S3).

The six topologies were exported to RStudio v1.1.383 [34] running as an IDE for R v3.4.2 [35], with all polytomies randomly dichotomized using the function *multi2di* from the R package *ape* v4.1 [36] (Fig. 1b). Branch lengths were calibrated using the reported geological age of each specimen in the corresponding publication or the compiled geological ages available in the Paleobiology Database (PaleoDB, https://paleobiodb.org/). If no data on geological age was available for a given specimen, we used either the sister taxon’s age or the complete clade age range (see supplementary methods in the Additional file 1). These age ranges were then used in the *cal3* algorithm of the R package *paleotree* v2.7 [37–39] (Fig. 1c). This algorithm calibrates branch lengths using a stochastic sampling of node ages under a birth-death-sampling model; for this we used the theropod sampling rate reported in [40] and used it to estimate the extinction rate. Diversification rate was assumed to be equal to the extinction rate, which has been argued to be a reasonable assumption for extinct clades [39]. After dichotomization and calibration 1 was added to each branch length in order to maintain the node structure derived from the base topologies. The use of geological age for branch calibration is a common methodological approach when dealing with data from the fossil record [22, 41–43], although there are many ways in which this method can create bias as a result of missing information. However, our results did not show significant changes when using a “null” model of uniform branch length (not shown), showing them to be robust to branch length variation.

For those analyses considering the complete dataset, including multiple specimens per species, we adapted the method described in [44] for Phylogenetically Independent Contrasts (PIC) and used it for Phylogenetic Generalized Least Squares (PGLS) [45], looking to maximize the shared evolutionary history represented in the trees for conspecifics, and thus minimizing their relative weight [46, 47]. Therefore, we replaced the tips of each species represented by more than a single specimen with a zero-branch length polytomy (Fig. 1d). However, it should be noted that this method assumes that every specimen of a species has the same geological age and ignores possible factors that may reduce the covariance between specimens.

### Regressions and statistical tests

Regressions were done using PGLS [48] under two different evolutionary models: Brownian Motion (BM) [49, 50] (reported as a model for body size evolution in Theropoda [21]); as well as Ornstein-Uhlenbeck (OU) [51, 52]. However, we advise caution when interpreting results with the latter, since it has been reported to incur in elevated type I errors when used for low sample sizes or low attraction strength (α) [52]. Regressions using BM were done with simultaneous estimation of phylogenetic signal using Pagel’s λ [46, 53, 54], which is a coefficient that downweighs the covariance matrix derived of the phylogenetic trees, according to how closely does the observed trait distribution resemble one simulated under pure Brownian Motion (BM). In this sense a λ = 0 implies a trait distribution completely independent of the phylogeny and λ = 1 one that exactly mirrors what would be expected had the trait evolved under BM. For the regressions we used the R packages *nlme* v3.1 [55] and *ape* v4.1 [36]. When λ estimation was not possible a fixed value of λ = 1 was assigned and an additional regression using Ordinary Least Squares (OLS) was done, which is the equivalent of performing a PGLS with λ = 0, in order to assess the full range of possible values. We performed the regressions on each of the 6000 dichotomous and calibrated trees (1000 per topology) and then, the estimated parameters were pooled using multiple imputation methods [56, 57] for each evolutionary model. It is worth noting that these methods converge to the one presented in [58] when extended to all the possible trees. Parameter pooling was done using the R package *mice* v2.30 [59] and pooled parameters were estimated for each topology plus a total pool considering all 6000 regressions. We also performed regressions for each clade within Theropoda using PGLS in order to evaluate clade-specific allometric patterns. All these analyses were carried out twice for each model: once using the complete dataset (including intraspecific variability) and once with the reduced dataset (including only a single largest specimen per species, see Additional file 1: Tables S5 for BM and S14 for OU). We also used PGLS to perform regressions on the dataset of Theropoda that excluded juvenile specimens, as well as the dataset composed only by specimens proposed to be juveniles.

To test whether any specific subclades significantly deviate from the main allometric trend of Theropoda, we used Phylogenetic ANCOVA (PANCOVA) [60]. Starting with the complete dataset, we tested for differences in Scansoriopterygidae, Troodontidae, Dromaeosauridae, Oviraptorosauria, Therizinosauria, Ornithomimosauria, Compsognathidae, Tyrannosauroidea, Megaraptora, Megalosauroidea, Ceratosauria, Coelophysoidea and the paraphyletic *Anchiornis*-related taxa. However, because most of these subclades are represented by few specimens, results must be considered as a first approach to identifying those sublcades that are most likely to show a different trend. PANCOVA tests were performed over a subset of 200 trees for each topology due to computational limitations, and the resulting *p*-values were then pooled using a meta-analysis method for non-independent tests [61]. Taxa consistently showing significant deviations from the main allometric trend for Theropoda across all topologies were then excluded and a new set of regressions were carried out. This process of regressions and PANCOVA testing was repeated in an iterative fashion until no new taxa were consistently excluded.

## Results

### Main Allometric trends and outliers

**Fig. 2:**
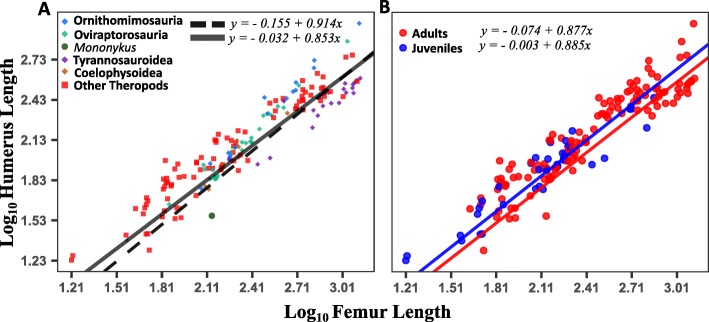
PGLS regressions under BM for humeral against femoral measurements, (**a**) regressions for the complete dataset before (dotted line) and after (continuous line) excluding subclades that showed significant differences from the main allometric trend after Phylogenetic ANCOVA testing (Oviraptorosauria, Ornithomimosauria, Tyrannosauroidea, Coelophysoidea and Mononykus), (**b**) regressions for adult (red) and juvenile (blue) specimens

Our general analysis (complete dataset of all Theropoda) shows negative forelimb allometry that is significantly different from isometry for both BM and OU models (BM: 0.914, 95% CI [0.864, 0.964], λ = 0.930, Fig. 2a; OU: 0.941, 95% CI [0.900, 0.982], α = 0.013, Additional file 1: Figure S1). The estimated parameters for the reduced dataset (with only one specimen per species) also showed negative forelimb allometry that is significantly different from isometry (BM: 0.901, 95% CI [0.843, 0.959], λ = 0.954; OU: 0.906, 95% CI [0.848, 0.965], α = 0.013). It should be noted that no statistically significant difference was found between the allometric coefficients estimated for the complete and reduced dataset. Thus, we discarded any concerns about over-representation of species with multiple specimens. Allometric coefficients and λ or α estimations for the pooled regressions of topologies 1 through 6 resulted in similar values for BM and OU respectively (Additional file 1: Tables S1 and S2 for BM, Tables S7 and S8 for OU), therefore the total pooled coefficient and λ or α, considering all 6000 trees across the six topologies, properly represents the mean estimated parameters for all the considered phylogenetic hypotheses.

Estimated parameters for the dataset comprising only adult specimens (BM: 0.877, 95% CI [0.808, 0.945], λ = 0.944; OU: 0.877, 95% CI [0.758, 0.996], α = 0.493) showed no statistical difference when compared to those of our general analysis. Also, a dataset comprised only of specimens proposed to be juveniles resulted in similar parameters to adults under BM (0.885, 95% CI [0.798, 0.973], λ = 0.949), differing only on their intercepts (Fig. 2b), with higher differences found under OU (0.938, 95% CI [0.839, 1.037], α = 0.007). However, under OU, low α values and low sample size (37 specimens) might make these parameters unreliable [52]. Therefore, we found no evidence to exclude juvenile specimens.

Upon using PANCOVA to test for clade-specific deviations from the main trend of negative forelimb allometry, we found that Oviraptorosauria, Ornithomimosauria, and Coelophysoidea showed significant differences consistently across all topologies under both BM and OU, with the addition of Tyrannosauroidea only under BM (Additional file 1: Table S3) and Scansoriopterygidae and Megalosauroidea only under OU (Additional file 1: Tables S9 and S10). We then carried out a new analysis with all these subclades excluded, to test whether any additional subclades deviate from the main trend. We also decided to exclude the alvarezsaurid *Mononykus olecranus*, a small-sized specimen with short forelimbs*,* whose position in charts is clearly offset from the distribution of most specimens (Fig. 2a). Although testing through phylogenetic ANCOVA cannot be carried out for a single data point, it is very likely that *Mononykus* does not conform to the main trend of negative allometry, not only because of its proportionally small forelimbs, but also because *Mononykus* evolved unique and extreme morpho-functional specialization of the forelimb, unlike any other theropods in our dataset (including its closest relative *Haplocheirus*). *Mononykus* has short, robust and highly muscularized forelimbs, and a monodactyl hand presenting a uniquely enlarged digit and claw. Along with a short radius and ulna and large olecranon process, the short and stout humerus has large muscle insertion sites, associated to a keeled and ossified sternum. This makes the forelimbs functionally comparable to those of digging tetrapods, which combined with the toothless and very slender snout suggest specialization in feeding on small insects [62].

Regressions on the remaining theropods resulted in lower allometric coefficients for both the complete (Fig. 2a) and reduced datasets, resulting in similar values for all topologies under BM (Table 1) and OU (Additional file 1: Table S12) with all allometric coefficients showing negative allometry and significant difference from isometry. After re-testing for deviations from the main forelimb allometric trend on the remaining subclades using PANCOVA, some additional taxa showed significant differences, but this depended on the topology used: No taxa presented significant differences across all six topologies (Additional file 1: Table S4 for BM and supplementary Table S11 for OU). Our analysis did not consistently support a different trend for Therizinosauria, which had been suggested to be outliers [11].

**Table 1:** Linear regression values for forelimb allometry for Theropoda under BM after excluding Oviraptorosauria, Ornithomimosauria, Tyrannosauroidea and Coelophysoidea

Complete dataset					Reduced dataset			
Topology	Intercept	Slope	Slope 95% CI	λ	Intercept	Slope	Slope 95% CI	λ
1	0.026	0.859	(0.797, 0.922)	0.939	0.079	0.840	(0.772, 0.908)	0.892
2	0.011	0.862	(0.800, 0.923)	0.939	0.068	0.840	(0.773, 0.907)	0.877
3	0.035	0.849	(0.787, 0.912)	0.938	0.092	0.831	(0.763, 0.899)	0.878
4	0.019	0.851	(0.789, 0.914	0.936	0.081	0.831	(0.764, 0.898)	0.860
5	0.057	0.848	(0.784, 0.911)	0.937	0.115	0.827	(0.761, 0.893)	0.875
6	0.043	0.849	(0.785, 0.913)	0.936	0.108	0.827	(0.762, 0.892)	0.848
1–6	0.032	0.853	(0.789, 0.917)	0.938	0.091	0.8336	(0.765, 0.900)	0.872

Intercept, Slope and Slope 95% CI are estimations obtained after pooling 1000 dichotomous time-scaled trees generated for the specified topology or topologies. λ is a simple mean of Pagel’s λ estimations on the same regressions

### Allometric trends within Theropod subclades

PGLS regressions for each clade largely confirmed the results obtained through PANCOVA: allometric coefficients estimated for Coelophysoidea, Ornithomimosauria and Oviraptorosauria resulted in isometry or values very near it, and no significant difference was observed with the reduced dataset, discarding the possibility that these trends could be due to overrepresentation of any single species (Table 2, Additional file 1: Table S5 for BM and supplementary Tables S13 and S14 for OU). For Oviraptorosauria the allometric coefficient recovered was similar to that reported in previous studies (1.014 for 12 specimens [63]).

**Table 2:** Pooled linear regressions under BM using the complete dataset for specific subclades

Clade	N	Top.	Intercept	Slope	Slope 95% CI	λ
Scansoriopterygidae	4	1–6	−0.053	1.073	(0.741, 1.404)	1^a^
		–	−0.075	1.085	(0.527, 1.644)	0^a^
Troodontidae + *Anchiornis* related	20	1–2	0.356	0.742	(0.520, 0.964)	1^a^
		3–4	0.352	0.743	(0.521, 0.964)	1^a^
		–	0.685	0.577	(0.380, 0.774)	0^a^
*Anchiornis* related	11	1–6	0.067	0.928	(0.723, 1.132)	1^a^
		–	0.025	0.962	(0.659, 1.264)	0^a^
Dromaeosauridae+ Troodontidae	31	1–2	0.144	0.833	(0.691, 0.975)	0.813
		3–4	0.133	0.839	(0.695, 0.983)	0.813
		5–6	0.173	0.832	(0.692, 0.971)	0.819
Troodontidae	9	1–6	0.300	0.739	(0.436, 1.042)	1^a^
		–	0.186	0.797	(0.479, 1.115)	0^a^
Dromaeosauridae	22	1–4	0.328	0.757	(0.597, 0.917)	1^a^
		5–6	0.399	0.728	(0.567, 0.888)	1^a^
		–	0.279	0.775	(0.732, 0.819)	0^a^
Dromaeosauridae excluding *Microraptor*	15	1–4	0.410	0.721	(0.523, 0.919)	1^a^
		5–6	0.471	0.697	(0.503, 0.890)	1^a^
		–	0.279	0.775	(0.732, 0.819)	0^a^
Oviraptorosauria	28	1–4	−0.204	0.988	(0.936, 1.041)	0.947
		5–6	−0.189	0.987	(0.930, 1.043)	0.946
Therizinosauria	5	1–6	0.062	0.912	(0.755, 1.070)	1^a^
		–	0.217	0.849	(0.587, 1.111)	0^a^
Ornithomimosauria	15	1–6	−0.348	1.057	(0.928, 1.186)	1^a^
		–	−0.543	1.143	(1.040, 1.245)	0^a^
Compsognathidae	7	1–6	−0.042	0.876	(0.580, 1.172)	0.717
Tyrannosauroidea	23	1–6	0.018	0.859	(0.650, 1.068)	0.437
Tyrann. + Megaraptoridae	25	2, 4, 6	−0.0171	0.882	(0.709, 1.054)	0.772
Allo. + Megaraptora	8	1, 3, 5	0.424	0.719	(0.266, 1.172)	1^a^
		–	0.497	0.695	(0.206, 1.184)	0^a^
Megalosauroidea	5	1–6	−0.258	0.988	(0.930, 1.046)	1^a^
		–	−0.268	0.991	(0.909, 1.073)	0^a^
Ceratosauria	9	1–6	0.276	0.763	(0.642, 0.884)	0.930
Coelophysoidea	10	1–6	−0.463	1.073	(0.863, 1.282)	1^a^
		–	−0.407	1.054	(0.842, 1.265)	0^a^
Non-Maniraptoriform Tetanurae	44	1–6	−0.047	0.896	(0.811, 0.980)	0.763
Non-Maniraptoriform Tetanurae excluding Tyrann.	21	1–6	−0.055	0.908	(0.815, 1.000)	0.753

N is the number of specimens, Intercept, Slope and Slope 95% CI are estimations obtained after pooling 1000 dichotomous time-scaled trees generated for the specified topologies. λ is a simple mean of Pagel’s λ estimations on the same regressions. ^a^denotes fixed λ values. *Top*. Topologies, *Tyrann*. Tyrannosauroidea, *Allo*. Allosauroidea

In Tyrannosauroidea, a clade that was also found to be significantly different under BM, there is a lower value of negative allometry than the main allometric trend for Theropoda, but a wide confidence interval, with no significant difference from isometry. Given that the allometric coefficient of Tyrannosauroidea is within what could be expected for other theropods, we performed a new PANCOVA in order to test if this clade was initially shown to be significantly different due to the influence of Coelophysoidea, Ornithomimosauria and Oviraptorosauria pulling the main theropod allometric coefficient towards higher values. The results of this test once again showed significant differences between Tyrannosauroidea and the rest of the dataset (Additional file 1: Table S6).

**Fig. 3:**
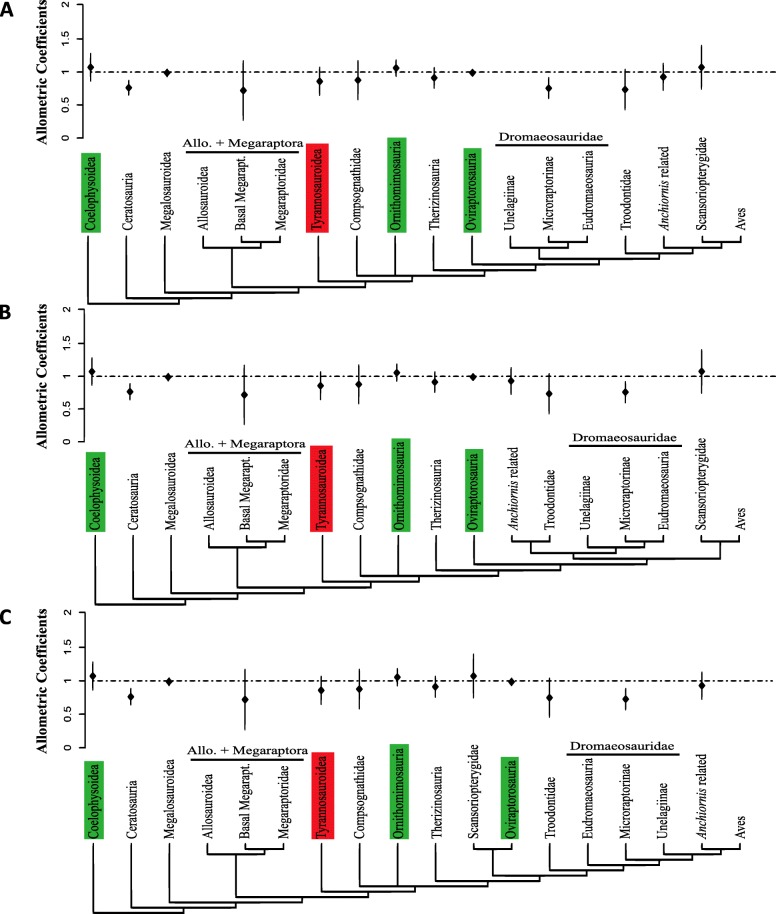
Clade specific allometric coefficients under BM for topologies (**a**) 1, (**b**) 3 and (**c**) 5, bars indicate 95% confidence interval. The subclades that showed significant differences from the main allometric trend of Theropoda through Phylogenetic ANCOVA testing are marked in green (isometry) and red (negative allometry)

The case of Scansoriopterygidae is peculiar: its allometric coefficient indicates isometry, however, the confidence interval is so wide that it doesn’t allow us to rule out either strong negative or positive allometries (Table 2, Fig. 3, Additional file 1: Tables S5. S13 and S14). Similarly, wide confidence intervals for allometric coefficients are found in Troodontidae, Compsognathidae and the subclade containing Allosauroidea and Megaraptoridae (only possible in topologies 1,3 and 5), although in these subclades, the upper limit of the confidence interval is near isometric, rather than one of positive allometry (Table 2. Figure 3, Additional file 1: Tables S5, S13 and S14). Wide confidence intervals in Scansoripterygidae could result from the very low sample size, although other subclades with similar sample sizes such as Therizinosauria and Megalosauroidea show narrower confidence intervals.

In subclades that are proposed as close relatives of Aves, we evaluated the allometric coefficient of *Anchiornis-*related taxa, Troodontidae, and Dromaeosauridae, as well as combinations of these taxa (Dromaeosauridae + Troodontidae for all topologies and Troodontidae + *Anchiornis*-related taxa in topologies 1–4). Scansoriopterygidae was considered, although some authors have argued they are not among the closest relatives of Aves [17]. The *Anchiornis*-related taxa and Troodontidae show coefficients of negative allometry, but these are not significantly different from isometry. However, when grouped together (topologies 1–2 as a paraphyletic group and topologies 3–4 as a monophyletic clade) the resulting allometric coefficients of this clade showed strong negative allometry, significantly different from isometry.

Dromaeosauridae showed strong negative allometry under BM both alone and when grouped with Troodontidae, being significantly different from isometry in all cases. Similar values were found after removing the *Microraptor* specimens, due to them being possible outliers, given their flight adaptations (Table 2). Under OU Dromaeosauridae showed wider confidence intervals, where the upper limit reaches isometry (Additional file 1: Tables S13 and S14).

## Discussion

As assessed from the total dataset (without excluding any outliers), we confirm support for a main trend of negative forelimb allometry in the evolution of non-avian Theropoda [10, 11]. By testing for the presence of outliers, we have also provided formal support for the notion that some subclades likely presented a different trend in arm size evolution. However, upon excluding these outliers, further rounds of PANCOVA testing did not consistently identify any additional subclades as outliers. Had we continued until most subclades were separated, this would have supported a scenario of “total diversity”, where each clade has its own distinctive trend. Instead, our data suggests a scenario in which negative allometry represents the primitive or “default” trend for Theropoda, and subclades with different trends (“outliers”) are derived (see below for further discussion on the basal Coelophysoidea). This is consistent with the main trend recovered for the complete dataset, and the observation that larger animals with proportionally smaller forelimbs are present across several subclades.

Although the evolutionary trend of negative allometry has been discussed by some authors, these have offered few explanations about its possible underlying causes. The existence of such a well-defined macroevolutionary trend defies explanation of forelimb proportions as multiple independent events of adaptation. In this regard, allometric trends in evolution often reflect ontogenetic allometries, such that evolutionary changes in adult body size result in new proportions, as expected according to the pre-existing ontogenetic trends [64–66]. Therefore, an evolutionary trend of negative allometry could reflect a negative allometry in the ontogeny of non-avian theropods, namely, slower growth of the forelimbs [10]. Unlike non-avian theropods, in birds there is an opposite evolutionary trend of positive forelimb allometry, such that larger species have proportionally larger forelimbs [12]. Accordingly, positive allometric growth of the forelimbs has been documented in the ontogeny of several birds [67, 68] and has been explicitly discussed for *Oceanodroma leucorhoa* [69], *Larus californicus* [70] and *Anous minutus* [71]. Modern birds present an ample diversity of relative limb size proportions and locomotor strategies, which can also vary during ontogeny [67]. In some species, the phase of increased forelimb growth occurs at a later ontogenetic stage; for example, in *Anas platyrhynchos*, negative allometric growth of the forelimb occurs first; forelimb growth only increases at later stages, as forelimbs become relevant in jumping, swimming, landing, and finally flight [72]. Similarly, in *Vanellus vanellus,* there is a marked delay of forelimb growth during early ontogenetic stages, when juveniles are terrestrial and have well developed hindlimbs. Forelimb growth increases thereafter, as they become involved in flight [73]. In other species such as the Emu *Dromaius novahollandiae,* hatchlings are born with reduced forelimbs [68], but maintain little activity and growth up to the flightless adult, which presents reduced wings. Based on these observations, we propose that patterns of allometric forelimb growth in modern birds are related to their degree of functional activity (mostly locomotor) at different ontogenetic stages. This suggests that putative negative allometry in the ontogeny of theropod dinosaurs could be related to an important phase of decreased function and activity of the forelimb.

The possibility of slower forelimb growth in the ontogeny of any non-avian dinosaur was first brought up by Gould and Lewontin, who were not satisfied with adaptive explanations for marked forelimb reduction in *Tyrannosaurus* [74]. Instead, they proposed that forelimb reduction was not an adaptation but “a developmental correlate of allometric fields for relative increase in head and hindlimb size”, a morphodynamic view that has been later expanded upon [75]. Slower growth of the forelimb in non-avian theropods is an explicit hypothesis, but fossils of juvenile specimens that can be confidently assigned to a given genus (let alone species) are rare. The best formal attempt has been carried out in *Allosaurus*, for which large concentrations of disarticulated remains have been found. Numerous femora and humeri representing different ontogenetic stages have been studied using LAGs (Lines of Arrested Growth) to estimate the age of each element. This uncovered negative allometric growth of the humerus [76], as expected within our interpretation. Ontogenetic negative allometry has been argued for humerus length in Tyrannosauridae [77], although this analysis grouped specimens from different genera, and the juvenile status of some specimens has been questioned [78]. No other studies have provided any statistical assessment of negative forelimb allometry in the ontogeny of non-avian theropods. In the data set used in our study, some genera were represented by multiple specimens exhibiting body size variation, which arguably could comprise specimens at different ontogenetic stages. Although we did not carry out an independent assessment of ontogenetic stages, we carried out regressions within individual genera and species, as a preliminary assessment of potential ontogenetic trends. In most cases, these regressions did not provide statistical support to either confirm or discard a potential ontogenetic allometry (Additional file 1: Table S15), which is probably due to reduced sample sizes and/or non-ontogenetic variation. The exception was *Coelophysis bauri*, which presented a potential ontogenetic trend of positive allometry, but this trend was not significantly different from isometry when analyzing *Coelophysis* as a genus (*C. bauri + C. rhodesiensis*).

In non-theropod dinosaurs, studies of different ontogenetic stages are available for the ornithischian *Psittacosaurus lujiatunensis* [79] and for the basal sauropodomorphs *Massospondylus carinatus* [80], *Riojasaurus incertus* [81]*,* and *Mussaurus patagonicus* [82]. In all of these non-theropod dinosaurs, early stages show greater forelimb proportions. For some of these, it has been suggested that early stages presented quadrupedal locomotion, and then transitioned into bipedal locomotion and proportionally shorter forelimbs [79, 80]. Biomechanical studies of different ontogenetic stages of *Mussaurus* have now confirmed this quadrupedal-to-bipedal transition, showing how the center of mass was placed more anteriorly in younger individuals, and then shifted posteriorly as the tail grew proportionally larger and the neck became more slender [82]. The fact that the forelimbs experienced decreased growth along ontogeny was likely related to their decreased function in locomotion. The ontogenetic transition from quadrupedal hatchlings to bipedal adults has been suggested to be widespread among dinosaurs, and to represent the ancestral condition for this group [76]. In this regard, the absence of negative allometry in the evolution of Coelophysoidea (and possibly, in the ontogeny of *Coelophysis*) could be a derived condition. Alternatively, because Coelophysoidea is an outgroup to all other theropods in our analysis, it may represent the primitive trend for Theropoda. If so, negative allometry may have become the main trend shortly thereafter, in a slightly more exclusive clade approaching Averostra.

Many theropods could have presented an important ontogenetic phase of decreased forelimb growth, as suggested by the main evolutionary trend of negative allometry and the negative coefficients found in several subclades. Decreased forelimb growth in turn suggests decreased function along ontogeny. This decrease does not imply the absence of function: Most likely, some important function was performed at early stages, and was then lost in ontogeny, while other functions continued to be performed, such as those that have been well-discussed for adults (especially the manipulation and carrying of prey, [83–86]). The proposed phase of decreased forelimb growth may have extended through a substantial portion of ontogeny, as suggested by ontogenetic data from *Allosaurus* [76], and the fact that evolutionary negative allometry is observable across a broad range of body sizes (including the upper large/gigantic range). However, it is hard to infer how early in ontogeny could the onset of decreased forelimb growth occur. Upon hatching, the forelimbs could have first experienced an early burst of positive allometric growth (as in modern birds), only experiencing decreased growth thereafter. Alternatively, forelimbs may have already been proportionally large, as in the quadrupedal hatchlings of non-theropod dinosaurs. Fossil evidence from theropod hatchlings is sparse, and mostly inferred from near-hatching embryos that cannot be confidently assigned to an adult species. In Therizinosauria, embryos have been described as having forelimb and hindlimb elements of similar size, along with the suggestion that hatchlings were quadrupedal [87]. Although no measurements were provided, such forelimb proportions would be greater than any adult members of Therizinosauria, supporting ontogenetic negative allometry in this clade, and large forelimb proportions upon hatching. In Oviraptorosauria, a clade that deviates from the main trend of negative allometry, the forelimb proportions of embryos resemble those of adult members of this clade [88] suggesting ontogenetic isometry, as expected from their evolutionary trend.

More discussion about the ontogeny of forelimb growth and locomotion is available for theropods that are closer to birds, especially in relation to wing assisted locomotion and the origin of flight. The recent description of a juvenile specimen of *Deinonychus,* including partial remains of the forelimbs, shows that forelimbs were proportionally larger at early stages, but also suggests that they were functionally different from adults [89]. The presence of a well-developed olecranon process only in the juvenile specimen has been argued to allow for greater extension of the arm (in contrast with a more permanently flexed position of the elbow in the adult), suggesting some form of wing assisted locomotion [89]. This is reasonable considering the presence of large remigial feathers on the forearms of Pennaraptora (including Dromaeosauridae), and the fact that these feathers were well developed even before hatching in closely related basal Aves [90, 91]. The case of *Deinonychus* may be compared with that of the modern bird *Alectura lathami*, where wing assisted locomotion is more developed in juveniles than adults [92, 93]. For theropod taxa close to Aves, wing-assisted locomotion (such as incline running, or even flight at early, small body sizes) provides an important function that could have reasonably been lost along ontogeny, as body size increased. It is worth noting that our data confirms that Dromaeosauridae has a marked evolutionary trend of negative allometry, that is significantly different from isometry (Table 2, supplementary Tables S5, S13 and S14). Thus, despite evolving small volant taxa such as *Microraptor*, there was no switch to positive allometry as the main evolutionary trend, unlike the closely related Aves [94].

Theropods leading to Aves experienced a marked decrease in body size [6, 7] which is consistent with an accompanying trend of paedomorphosis in skull morphology [95, 96]. As such, besides traits such as an enlarged orbit and braincase, proportionally larger forelimbs could be another juvenile trait [10]. Forelimbs of paedomorphic adults would have remained functional, omitting the later stages of decreased function and negative allometric growth. In this regard, it is worth noting that both Ornithomimosauria and Oviraptorosauria show bird-like skulls with arguably paedomorphic traits, which also coincides with the loss of negative forelimb allometry as an evolutionary trend in these subclades (see the supplementary discussion in Additional file 1). An intriguing possibility is that in the lineage leading to birds, wing-assisted locomotion first evolved in juveniles with proportionally larger forelimbs, perhaps as a mechanism of predator avoidance, and only later became an adult trait in smaller paedomorphic forms. Hopefully, new fossil evidence will provide increasingly detailed information about forelimb ontogeny along the dinosaur-bird transition.

## Conclusions

Our results confirm that negative forelimb allometry is the main evolutionary trend for non-avian theropods. Through Phylogenetic ANCOVA testing, we also identified that Coelophysoidea, Ornithomimosauria and Oviraptorosauria deviate from the main trend of negative allometry, with support for evolutionary trends closer to isometry.

Evolutionary allometric trends often reflect ontogenetic allometries, which suggests an important stage of negative allometric growth of the forelimb during the ontogeny of most non-avian theropods. Accordingly, a different ontogenetic trend can be expected for those subclades with evolutionary trends that deviate from negative forelimb allometry. In modern birds, allometric growth of the limbs is related to locomotor and behavioral changes along ontogeny. Fossil evidence supports slower growth of the forelimb during ontogeny as an ancestral condition for dinosaurs, likely related to bipedism and decreased forelimb use. In subclades closer to Aves, such as Dromeosauridae, early ontogenetic stages may have used their forelimbs in wing assisted-locomotion, that was lost at later ontogenetic stages, as body size increased. We propose that proportionally longer arms of juveniles became adult traits in the small-sized and paedomorphic Aves.

## Supplementary information

**Additional file 1.** Supplementary Methods, Tables and Figures

**Additional file 2.** Dataset used for analysis

## Data Availability

Dataset used in the present work is available in Additional file 2.

## Abbreviations

BM: Brownian Motion
FL: Femoral Length
HL: Humeral Length
OLS: Ordinary Least Squares
OU: Ornstein-Uhlenbeck
PANCOVA: Phylogenetic ANCOVA
PGLS: Phylogenetic Generalized Least Squares
PIC: Phylogenetic Independent Contrasts
