# Supplementary material for: Assesment and interpretation of negative forelimb allometry in the evolution of non-avian Theropoda
Source: Front Zool. 2019 Dec 2;16:44. doi: 10.1186/s12983-019-0342-9 (PMC6889632; doi:10.1186/s12983-019-0342-9)

Additional file 1 for

**Assesment and Interpretation of Negative Forelimb Allometry in The Evolution of Non-Avian Theropoda**

*José Palma Liberona^1,*^, Sergio Soto-Acuña^1^, Marco A. Mendez^2^, Alexander O. Vargas^1,^*^*^

1. Laboratorio de Ontogenia y Filogenia, Departamento de Biología, Facultad de Ciencias, Universidad de Chile. Las Palmeras 3425, Santiago.

2. Laboratorio de Genética y Evolución, Departamento de Ciencias Ecológicas, Facultad de Ciencias, Universidad de Chile. Las Palmeras 3425, Santiago.

*Corresponding authors [jpalmaliberona@gmail.com](mailto:jpalmaliberona@gmail.com); [alexvargas@uchile.cl](mailto:alexvargas@uchile.cl)

**This file includes:**

Supplementary methods

Supplementary Tables S1-S15

Supplementary discussion of Table S15

Supplementary Figure S1-S3

**SUPPLEMENTARY METHODS**

**1.- Age ranges for specimens with missing age estimation**

No age information was found for *Daliansaurus* (Shen *et al.*, 2017), *Jianhuanlong* (Xu *et al*., 2017), *Zhenyuanlong* (Lü and Brussate 2015), the dromaeosauridae NGMC 91 (Ji *et al.*, 2001), the Oviraptoridae MPC 1 (measures taken from Fanti *et al.*, 2012), *Citipati sp*. IGM 100/42 and *Richenia* (Fanti *et al.*, 2012). In order to provide age ranges for these specimens we assigned them ages depending their positions: specimens in fully resolved nodes were assigned the age range of their sister taxon; specimens in polytomies were assigned the complete age range corresponding to all other specimens in the politomy; finally, specimens in unresolved positions basal to a clade were assigned the complete range of ages corresponding to the whole clade.

**2.- Intraspecific regressions**

Simple non phylogenetic Ordinary Least Squares regressions where performed for each genus and species represented in our dataset by 3 or more individuals. Results are shown in supplementary table S15.

**SUPPLEMENTARY TABLES**

**Table S1:** Linear regression values for forelimb allometry for Theropoda using the complete dataset under BM. Intercept, Intercept Standard Deviation (I SD), Slope, Slope Standard Deviation (S SD) and Slope 95% CI are estimations obtained after pooling 1000 dichotomous time-scaled trees generated for the specified topology or topologies. λ is a simple mean of Pagel’s λ estimations on the same regressions.

| Topology | Intercept | I SD | slope | S SD | slope CI_min | slope CI_max | λ |
| --- | --- | --- | --- | --- | --- | --- | --- |
| Topology 1 | -0.1625 | 0.0965 | 0.9208 | 0.0247 | 0.8724 | 0.9693 | 0.9287 |
| Topology 2 | -0.1733 | 0.0980 | 0.9223 | 0.0247 | 0.8738 | 0.9708 | 0.9299 |
| Topology 3 | -0.1557 | 0.0988 | 0.9120 | 0.0249 | 0.8632 | 0.9609 | 0.9301 |
| Topology 4 | -0.1700 | 0.0995 | 0.9145 | 0.0249 | 0.8656 | 0.9634 | 0.9310 |
| Topology 5 | -0.1281 | 0.0978 | 0.9066 | 0.0247 | 0.8581 | 0.9551 | 0.9292 |
| Topology 6 | -0.1407 | 0.0988 | 0.9088 | 0.0248 | 0.8603 | 0.9574 | 0.9304 |
| Topologies 1-6 | -0.1551 | 0.0995 | 0.9142 | 0.0255 | 0.8643 | 0.9641 | 0.9299 |

**Table S2:** Linear regression values for forelimb allometry for Theropoda using the reduced dataset under BM. Intercept, Intercept Standard Deviation (I SD), Slope, Slope Standard Deviation (S SD) and Slope 95% CI are estimations obtained after pooling 1000 dichotomous time-scaled trees generated for the specified topology or topologies. λ is a simple mean of Pagel’s λ estimations on the same regressions.

| Topology | Intercept | I SD | Slope | S SD | Slope CI_min | Slope CI_max | λ |
| --- | --- | --- | --- | --- | --- | --- | --- |
| Topology 1 | -0.1284 | 0.1058 | 0.9091 | 0.0285 | 0.8532 | 0.9649 | 0.9541 |
| Topology 2 | -0.1418 | 0.1073 | 0.9119 | 0.0287 | 0.8556 | 0.9681 | 0.9524 |
| Topology 3 | -0.1159 | 0.1084 | 0.8981 | 0.0289 | 0.8415 | 0.9548 | 0.9527 |
| Topology 4 | -0.1328 | 0.1090 | 0.9019 | 0.0290 | 0.8451 | 0.9587 | 0.9492 |
| Topology 5 | -0.0849 | 0.1079 | 0.8919 | 0.0285 | 0.8360 | 0.9477 | 0.9585 |
| Topology 6 | -0.1004 | 0.1088 | 0.8953 | 0.0288 | 0.8389 | 0.9516 | 0.9545 |
| Topologies 1-6 | -0.1174 | 0.1096 | 0.9013 | 0.0296 | 0.8433 | 0.9594 | 0.9536 |

**Table S3:** p-values for phylogenetic ANCOVA under BM performed over the complete dataset, we used values < 0.05 as a cut point (shown in bold), the analysis was performed in a subset of 200 time calibrated trees for each topology. T 1-6 pool indicates the value of the pooled p-vals of the all the tests done on topologies 1 through 6.

|  | Topology 1 | Topology 2 | Topology 3 | Topology 4 | Topology 5 | Topology 6 | T 1-6 pool |
| --- | --- | --- | --- | --- | --- | --- | --- |
| Scansoriopterygidae | 0.4032 | 0.4209 | 0.4803 | 0.4439 | 0.4325 | 0.4332 | 0.4355 |
| Anchiornis related | 0.9646 | 0.9668 | 0.9693 | 0.9638 | 0.9708 | 0.9656 | 0.9672 |
| Troodontidae | 0.5318 | 0.8399 | 0.4890 | 0.7554 | 0.4864 | 0.7570 | 0.6350 |
| Dromaeosauridae | 0.4005 | 0.3634 | 0.3937 | 0.4233 | 0.3667 | 0.4281 | 0.3956 |
| Oviraptorosauria | **0.0482** | **0.0486** | **0.0301** | **0.0348** | **0.0000** | **0.0000** | **0.0000** |
| Therizinosauria | 0.9145 | 0.9156 | 0.9617 | 0.9648 | 0.9708 | 0.9675 | 0.9509 |
| Ornithomimosauria | **0.0000** | **0.0000** | **0.0026** | **0.0022** | **0.0000** | **0.0000** | **0.0000** |
| Compsognathidae | 0.3604 | 0.3474 | 0.3487 | 0.3257 | 0.4434 | 0.4285 | 0.3737 |
| Tyrannosauroidea | **0.0115** | **0.0127** | **0.0056** | **0.0060** | **0.0089** | **0.0097** | **0.0085** |
| Megaraptora | 0.6436 | 0.6643 | 0.6201 | 0.6330 | 0.6215 | 0.6351 | 0.6370 |
| Megalosauroidea | 0.2771 | 0.2696 | 0.3523 | 0.3368 | 0.3562 | 0.3344 | 0.3194 |
| Ceratosauria | 0.6564 | 0.6619 | 0.5797 | 0.5882 | 0.5761 | 0.5877 | 0.6084 |
| Coelophysoidea | **0.0142** | **0.0137** | **0.0165** | **0.0166** | **0.0166** | **0.0159** | **0.0154** |

**Table S4:** p-values for phylogenetic ANCOVA under BM performed over the remaining theropods once Oviraptorosauria, Ornithomimosauria, Tyrannosauroidea and Coelophysoidea were excluded. We used values < 0.05 as a cut point (shown in bold), T 1-6 pool indicates the value of the pooled p-vals of the all the tests done on topologies 1 through 6.

|  | Topology 1 | Topology 2 | Topology 3 | Topoloy 4 | Topology 5 | Topology 6 | T 1-6 pool |
| --- | --- | --- | --- | --- | --- | --- | --- |
| Scansoriopterygidae | **0.0000** | **0.0000** | **0.0000** | **0.0000** | 0.4312 | 0.4967 | **0.0000** |
| Anchiornis related | **0.0000** | **0.0000** | **0.0000** | **0.0000** | 0.9718 | 0.9684 | **0.0000** |
| Troodontidae | **0.0000** | **0.0000** | **0.0000** | **0.0000** | 0.4923 | 0.7916 | **0.0000** |
| Dromaeosauridae | 0.3860 | **0.0000** | 0.3878 | **0.0000** | **0.0000** | **0.0000** | **0.0000** |
| Therizinosauria | **0.0000** | **0.0000** | **0.0000** | **0.0000** | 0.9717 | 0.9695 | **0.0000** |
| Compsognathidae | 0.3390 | **0.0000** | 0.3251 | **0.0000** | 0.0000 | **0.0000** | **0.0000** |
| Megaraptora | 0.6180 | 0.6588 | 0.6090 | **0.0000** | 0.5929 | **0.0000** | **0.0000** |
| Megalosauroidea | 0.2925 | 0.3102 | 0.3749 | **0.0000** | **0.0000** | **0.0000** | **0.0000** |
| Ceratosauria | 0.6114 | 0.6353 | 0.5351 | 0.5832 | **0.0000** | **0.0000** | **0.0000** |

**Table S5:** Pooled linear regressions using the reduced dataset for specific subclades under BM. N is the number of specimens, Intercept, Slope and Slope 95% CI are estimations obtained after pooling 1000 dichotomous time-scaled trees generated for the specified topology or topologies. λ is a simple mean of Pagel’s λ estimations on the same regressions. † denotes fixed λ values. Only subclades represented by more than three specimens were included in this table. Top. = Topologies, Tyrann. = Tyrannosauroidea, Allo. = Allosauroidea.

| Clade | N | Top. | Intercept | Slope | Slope 95% CI | λ |
| --- | --- | --- | --- | --- | --- | --- |
| Scansoriopterygidae | 3 | 1-6 | -0.054 | 1.074 | (0.623, 1.524) | 1^†^ |
|  |  | - | -0.060 | 1.077 | (-1.777, 3.931) | 0^†^ |
| Troodontidae + *Anchiornis* related | 13 | 1-2 | 0.286 | 0.782 | (0.457, 1.106) | 1^†^ |
|  |  | 3-4 | 0.286 | 0.781 | (0.460, 1.101) | 1^†^ |
|  |  | - | 0.623 | 0.603 | (0.382, 0.823) | 0^†^ |
| *Anchiornis* related | 5 | 1-6 | -0.220 | 1.078 | (0.768, 1.388) | 1^†^ |
|  |  | - | -0.146 | 1.038 | (0.586, 1.490) | 0^†^ |
| Dromaeosauridae + Troodontidae | 23 | 1-2 | 0.277 | 0.767 | (0.586, 0.948) | 0.912 |
|  |  | 3-4 | 0.270 | 0.772 | (0.585, 0.959) | 0.896 |
|  |  | 5-6 | 0.339 | 0.755 | (0.585, 0.925) | 0.930 |
| Troodontidae | 8 | 1-6 | 0.286 | 0.752 | (0.399, 1.104) | 1^†^ |
|  |  | - | 0.395 | 0.702 | (0.342, 1.062) | 0^†^ |
| Dromaeosauridae | 15 | 1-4 | 0.404 | 0.735 | (0.533, 0.937) | 1^†^ |
|  |  | 5-6 | 0.450 | 0.716 | (0.506, 0.925) | 1^†^ |
|  |  | - | 0.185 | 0.856 | (0.631, 1.081) | 0^†^ |
| Dromaeosauridae excluding *Microraptor* | 14 | 1-4 | 0.407 | 0.734 | (0.520, 0.948) | 1^†^ |
|  |  | 5-6 | 0.456 | 0.713 | (0.492, 0.935) | 1^†^ |
|  |  | - | 0.158 | 0.867 | (0.623, 1.111) | 0^†^ |
| Oviraptorosauria | 21 | 1-4 | -0.266 | 1.016 | (0.909, 1.122) | 1^†^ |
|  |  | 5-6 | -0.241 | 1.009 | (0.893, 1.125) | 1^†^ |
|  |  | - | -0.371 | 1.056 | (0.959, 1.154) | 0^†^ |
| Therizinosauria | 5 | 1-6 | 0.062 | 0.912 | (0.755, 1.070) | 1^†^ |
|  |  | - | 0.217 | 0.849 | (0.587, 1.111) | 0^†^ |
| Ornithomimosauria | 8 | 1-6 | -0.526 | 1.130 | (0.999, 1.261) | 1^†^ |
|  |  | - | -0.519 | 1.131 | (1.017, 1.245) | 0^†^ |
| Compsognathidae | 5 | 1-6 | 0.039 | 0.836 | (-0.082, 1.753) | 1^†^ |
|  |  | - | 0.061 | 0.819 | (0.295, 1.343) | 0^†^ |
| Tyrannosauroidea | 12 | 1-6 | -0.160 | 0.934 | (0.786, 1.082) | 1^†^ |
|  |  | - | 0.227 | 0.758 | (0.509, 1.007) | 0^†^ |
| Tyrann. + Megaraptora | 14 | 2, 4, 6 | -0.170 | 0.944 | (0.772, 1.117) | 1^†^ |
|  |  | - | 0.287 | 0.743 | (0.488, 0.998) | 0^†^ |
| Allo. + Megaraptora | 8 | 1, 3, 5 | 0.424 | 0.719 | (0.206, 1.184) | 1^†^ |
|  |  | - | 0.497 | 0.695 | (0.266, 1.172) | 0^†^ |
| Megalosauroidea | 5 | 1-6 | -0.258 | 0.988 | (0.809, 1.168) | 1^†^ |
|  |  | - | -0.268 | 0.991 | (0.909, 1.073) | 0^†^ |
| Ceratosauria | 7 | 1-6 | 0.336 | 0.740 | (0.541, 0.940) | 1^†^ |
|  |  | - | -0.024 | 0.868 | (0.502, 1.233) | 0^†^ |
| Coelophysoidea | 4 | 1-6 | -0.316 | 1.008 | (0.814, 1.202) | 1^†^ |
|  |  | - | -0.300 | 1.002 | (0.617, 1387) | 0^†^ |
| Non-Maniraptoriform Tetanurae | 31 | 1-6 | -0.119 | 0.918 | (0.826, 1.010) | 0.846 |
| Non-Maniraptoriform Tetanurae excluding Tyrann. | 19 | 1-6 | -0.068 | 0.905 | (0.787, 1.022) | 1^†^ |
|  |  | - | -0.110 | 0.915 | (0.840, 0.989) | 0^†^ |

**Table S6**

p-values for phylogenetic ANCOVA under BM performed over the remaining theropods once Trannosauroidea was reintegrated. We used values < 0.05 as a cut point (shown in bold), T 1-6 pool indicates the value of the pooled p-vals of the all the tests done on topologies 1 through 6.

|  | Topology 1 | Topology 2 | Topology 3 | Topology 4 | Topology 5 | Topology 6 | T 1-6 pool |
| --- | --- | --- | --- | --- | --- | --- | --- |
| Scansoriopterygidae | 0.4278 | 0.4350 | 0.4705 | 0.4688 | 0.4627 | 0.5022 | 0.4612 |
| Anchiornis related | 0.9607 | 0.9577 | 0.9659 | 0.9670 | 0.9684 | 0.9679 | 0.9652 |
| Troodontidae | 0.5207 | 0.8157 | 0.4758 | 0.7322 | 0.4698 | 0.7548 | 0.6187 |
| Dromaeosauridae | 0.3740 | 0.3503 | 0.3859 | 0.4322 | 0.3593 | 0.3913 | 0.3817 |
| Therizinosauria | 0.9034 | 0.9019 | 0.9546 | 0.9574 | 0.9669 | 0.9627 | 0.9430 |
| Compsognathidae | 0.3501 | 0.3345 | 0.3271 | 0.3249 | 0.4229 | 0.3953 | 0.3577 |
| Tyrannosauroidea | **0.0108** | **0.0121** | **0.0059** | **0.0000** | **0.0091** | **0.0099** | **0.0000** |
| Megaraptora | 0.6201 | 0.6376 | 0.5950 | 0.6142 | 0.6085 | 0.6007 | 0.6137 |
| Megalosauroidea | 0.2826 | 0.2816 | 0.3558 | 0.3489 | 0.3623 | 0.0000 | 0.0000 |
| Ceratosauria | 0.6109 | 0.6183 | 0.5476 | 0.5554 | 0.5441 | 0.5564 | 0.5725 |

**Table S7:** Linear regression values for forelimb allometry for Theropoda using the complete dataset under OU. Intercept, Intercept Standard Deviation (I SD), Slope, Slope Standard Deviation (S SD) and Slope 95% CI are estimations obtained after pooling 1000 dichotomous time-scaled trees generated for the specified topology or topologies. α is a simple mean of α estimations on the same regressions.

| Topology | Intercept | I SD | slope | S SD | slope CI_min | slope CI_max | α |
| --- | --- | --- | --- | --- | --- | --- | --- |
| Topology 1 | -0.1500 | 0.0589 | 0,9426 | 0,0207 | 0,9021 | 0,9831 | 0,0130 |
| Topology 2 | -0.1499 | 0.0608 | 0,9392 | 0,0209 | 0,8982 | 0,9802 | 0,0122 |
| Topology 3 | -0.1483 | 0.0594 | 0,9428 | 0,0207 | 0,9022 | 0,9834 | 0,0124 |
| Topology 4 | -0.1511 | 0.0614 | 0,9397 | 0,0210 | 0,8986 | 0,9809 | 0,0116 |
| Topology 5 | -0.1431 | 0.0610 | 0,9424 | 0,0205 | 0,9022 | 0,9826 | 0,0143 |
| Topology 6 | -0.1476 | 0.0609 | 0,9396 | 0,0205 | 0,8994 | 0,9797 | 0,0124 |
| Topologies 1-6 | -0.1483 | 0.0995 | 0,9411 | 0,0208 | 0,9003 | 0,9818 | 0,0126 |

**Table S8:** Linear regression values for forelimb allometry for Theropoda using the reduced dataset under OU. Intercept, Intercept Standard Deviation (I SD), Slope, Slope Standard Deviation (S SD) and Slope 95% CI are estimations obtained after pooling 1000 dichotomous time-scaled trees generated for the specified topology or topologies. α is a simple mean of α estimations on the same regressions.

| Topology | Intercept | I SD | Slope | S SD | Slope CI_min | Slope CI_max | α |
| --- | --- | --- | --- | --- | --- | --- | --- |
| Topology 1 | -0,0987 | 0,0854 | 0,9133 | 0,0288 | 0,8569 | 0,9697 | 0,0122 |
| Topology 2 | -0,1082 | 0,0861 | 0,9164 | 0,0290 | 0,8596 | 0,9731 | 0,0123 |
| Topology 3 | -0,0748 | 0,0851 | 0,9028 | 0,0292 | 0,8457 | 0,9600 | 0,0135 |
| Topology 4 | -0,0871 | 0,0854 | 0,9067 | 0,0293 | 0,8493 | 0,9640 | 0,0137 |
| Topology 5 | -0,0527 | 0,0859 | 0,8977 | 0,0293 | 0,8403 | 0,9551 | 0,0128 |
| Topology 6 | -0,0622 | 0,0864 | 0,9008 | 0,0294 | 0,8431 | 0,9584 | 0,0131 |
| Topologies 1-6 | -0,0806 | 0,0879 | 0,9063 | 0,0299 | 0,8477 | 0,9649 | 0,0129 |

**Table S9:** p-values for phylogenetic ANCOVA under OU performed over the complete dataset, we used values < 0.05 as a cut point (shown in bold), the analysis was performed in a subset of 200 time calibrated trees for each topology. T 1-6 pool indicates the value of the pooled p-vals of the all the tests done on topologies 1 through 6.

|  | Topology 1 | Topology 2 | Topology 3 | Topology 4 | Topology 5 | Topology 6 | T 1-6 pool |
| --- | --- | --- | --- | --- | --- | --- | --- |
| Scansoriopterygidae | **0,0274** | **0,0000** | **0,0302** | **0,0280** | **0,0000** | **0,0286** | **0,0000** |
| Anchiornis related | 0,7856 | 0,7793 | 0,7846 | 0,7737 | 0,7857 | 0,7804 | 0,7825 |
| Troodontidae | 0,9454 | 0,9619 | 0,9518 | 0,9775 | 0,9526 | 0,9806 | 0,9627 |
| Dromaeosauridae | 0,3941 | 0,4532 | 0,4212 | 0,4611 | 0,4187 | 0,4758 | 0,4370 |
| Oviraptorosauria | **0,0012** | **0,0011** | **0,0007** | **0,0006** | **0,0003** | **0,0003** | **0,0006** |
| Therizinosauria | 0,8798 | 0,8741 | 0,8538 | 0,8462 | 0,8604 | 0,8571 | 0,8628 |
| Ornithomimosauria | **0,0000** | **0,0000** | **0,0000** | **0,0000** | **0,0000** | **0,0000** | **0,0000** |
| Compsognathidae | 0,4197 | 0,4155 | 0,4251 | 0,4167 | 0,4547 | 0,4531 | 0,4309 |
| Tyrannosauroidea | 0,7010 | 0,7132 | 0,6861 | 0,7248 | 0,6912 | 0,7009 | 0,7037 |
| Megaraptora | 0,8406 | 0,8358 | 0,8397 | 0,8349 | 0,8393 | 0,8358 | 0,8386 |
| Megalosauroidea | 0,0759 | 0,0749 | 0,0757 | 0,0725 | 0,0766 | 0,0740 | 0,0746 |
| Ceratosauria | 0,6029 | 0,5941 | 0,6045 | 0,5903 | 0,6061 | 0,5981 | 0,6001 |
| Coelophysoidea | 0,0679 | 0,0635 | 0,0657 | 0,0609 | 0,0654 | 0,0639 | 0,0642 |

**Table S10:** p-values for phylogenetic ANCOVA under OU performed over the remaining theropods once Scansoriopterygidae, Oviraptorosauria and Ornithomimosauria, were excluded. We used values < 0.05 as a cut point (shown in bold), T 1-6 pool indicates the value of the pooled p-vals of the all the tests done on topologies 1 through 6.

|  | Topology 1 | Topology 2 | Topology 3 | Topoloy 4 | Topology 5 | Topology 6 | T 1-6 pool |
| --- | --- | --- | --- | --- | --- | --- | --- |
| Anchiornis related | 0,5201 | 0,5236 | 0,6069 | 0,5971 | 0,5295 | 0,5521 | 0,5548 |
| Troodontidae | 0,6186 | 0,6221 | 0,6633 | 0,6556 | 0,6228 | 0,6251 | 0,6353 |
| Dromaeosauridae | 0,2432 | 0,2247 | 0,3431 | 0,3066 | 0,2668 | 0,2559 | 0,2708 |
| Therizinosauria | 0,7333 | 0,7290 | 0,6401 | 0,6322 | 0,6484 | 0,6484 | 0,6722 |
| Compsognathidae | 0,3039 | 0,3023 | 0,3483 | 0,3394 | 0,4193 | 0,4161 | 0,3523 |
| Tyrannosauroidea | 0,9726 | 0,9722 | 0,9640 | 0,9698 | 0,9738 | 0,9686 | 0,9706 |
| Megaraptora | 0,7010 | 0,6964 | 0,7316 | 0,7280 | 0,7110 | 0,7084 | 0,7136 |
| Megalosauroidea | **0,0134** | **0,0137** | **0,0207** | **0,0198** | **0,0149** | **0,0154** | **0,0159** |
| Ceratosauria | 0,3454 | 0,3458 | 0,4000 | 0,3952 | 0,3582 | 0,3611 | 0,3672 |
| Coelophysoidea | **0,0181** | **0,0173** | **0,0251** | **0,0247** | **0,0178** | **0,0182** | **0,0198** |

**Table S11:**

p-values for phylogenetic ANCOVA under OU performed over the remaining theropods once Scansoriopterygidae, Oviraptorosauria, Ornithomimosauria, Megalosauroidea and Coelophysoidea were excluded. We used values < 0.05 as a cut point (shown in bold), T 1-6 pool indicates the value of the pooled p-vals of the all the tests done on topologies 1 through 6.

|  | Topology 1 | Topology 2 | Topology 3 | Topoloy 4 | Topology 5 | Topology 6 | T 1-6 pool |
| --- | --- | --- | --- | --- | --- | --- | --- |
| Anchiornis related | 0,4068 | 0,4055 | 0,3839 | 0,3690 | 0,3752 | **0,0000** | **0,0000** |
| Troodontidae | 0,7496 | 0,7695 | 0,7441 | 0,7412 | 0,7327 | **0,0000** | **0,0000** |
| Dromaeosauridae | 0,0875 | 0,0883 | 0,1467 | 0,1454 | 0,1333 | **0,0000** | **0,0000** |
| Therizinosauria | 0,6330 | 0,6301 | 0,6145 | 0,6112 | 0,6382 | **0,0000** | **0,0000** |
| Compsognathidae | 0,1606 | 0,1634 | 0,3123 | 0,3073 | 0,1824 | **0,0000** | **0,0000** |
| Tyrannosauroidea | 0,7577 | 0,7515 | 0,9424 | 0,9412 | 0,8455 | **0,0000** | **0,0000** |
| Megaraptora | 0,6853 | 0,6834 | 0,6827 | 0,6760 | 0,6753 | **0,0000** | **0,0000** |
| Ceratosauria | 0,2821 | 0,2847 | 0,2808 | 0,2834 | 0,2637 | **0,0000** | **0,0000** |

**Table S12:**

Linear regression values for forelimb allometry for Theropoda under OU after excluding Scansoriopterzgidae, Oviraptorosauria, Ornithomimosauria, Megalosauroidea and Coelophysoidea. Intercept, Slope and Slope 95% CI are estimations obtained after pooling 1000 dichotomous time-scaled trees generated for the specified topology or topologies. α is a simple mean of α estimations on the same regressions.

|  | Complete dataset | | | | Reduced dataset | | | |
| --- | --- | --- | --- | --- | --- | --- | --- | --- |
| Topology | Intercept | Slope | Slope 95% CI | α | Intercept | Slope | Slope 95% CI | α |
| 1 | 0,0296 | 0,8736 | (0,8135, 0,9336) | 0,0141 | 0,1021 | 0,8353 | (0,7614, 0,9093) | 0,0163 |
| 2 | 0,0389 | 0,8655 | (0,8021, 0,9289) | 0,0172 | 0,0885 | 0,8408 | (0,7657, 0,9158) | 0,0169 |
| 3 | 0,0333 | 0,8725 | (0,8124, 0,9326) | 0,0144 | 0,0929 | 0,8389 | (0,765, 0,9128) | 0,0159 |
| 4 | 0,0467 | 0,8626 | (0,798, 0,9272) | 0,0277 | 0,0782 | 0,8445 | (0,7694, 0,9197) | 0,0164 |
| 5 | 0,0319 | 0,8766 | (0,8159, 0,9374) | 0,0116 | 0,0868 | 0,8447 | (0,7707, 0,9186) | 0,0135 |
| 6 | 0,0721 | 0,8573 | (0,7566, 0,9581) | 0,3563 | 0,0713 | 0,8507 | (0,7754, 0,926) | 0,0139 |
| 1-6 | 0,0421 | 0,8680 | (0,797, 0,9391) | 0,0735 | 0,0866 | 0,8425 | (0,7673, 0,9176) | 0,0155 |

**Table S13:**

Pooled linear regressions using the complete dataset for specific subclades under OU. N is the number of specimens, Intercept, Slope and Slope 95% CI are estimations obtained after pooling 1000 dichotomous time-scaled trees generated for the specified topology or topologies. α is a simple mean of α estimations on the same regressions. Only subclades represented by more than three specimens were included in this table. Top. = Topologies, Tyrann. = Tyrannosauroidea, Allo. = Allosauroidea.

| Clade | N | Top. | Intercept | Slope | Slope 95% CI | α |
| --- | --- | --- | --- | --- | --- | --- |
| Scansoriopterygidae | 4 | 1-6 | -0,130 | 1,117 | (0.377, 1.858) | 2,882 |
| Troodontidae.+ *Anchiornis* related | 20 | 1-2 | 0,481 | 0,675 | (0.449, 0.901) | 1,048 |
|  |  | 3-4 | 0,470 | 0,681 | (0.434, 0.997) | 0,997 |
| *Anchiornis* related | 11 | 1-6 | 0,084 | 0,919 | (0.720, 1.118) | 1,175 |
| Dromaeosauridae + Troodontidae | 31 | 1-2 | 0,053 | 0,884 | (0.731, 1.037) | 0,768 |
|  |  | 3-4 | 0,054 | 0,882 | (0.728, 1.036) | 0,728 |
|  |  | 5-6 | 0,078 | 0,878 | (0.710, 1.047) | 0,752 |
| Troodontidae | 9 | 1-6 | 0,383 | 0,706 | (0.445, 0.967) | 0,923 |
| Droameosauridae | 22 | 1-4 | 0,144 | 0,867 | (0.703, 1.031) | 1,182 |
|  |  | 5-6 | 0,145 | 0,870 | (0.704, 1.037) | 1,092 |
| Dromaoesauridae excluding *Microraptor* | 15 | 1-4 | 0,163 | 0,859 | (0.619, 1.099) | 1,215 |
|  |  | 5-6 | 0,158 | 0,863 | (0.621, 1.106) | 1,113 |
| Oviraptorosauria | 28 | 1-4 | -0,248 | 1,006 | (0.936, 1.075) | 0,341 |
|  |  | 5-6 | -0,239 | 1,002 | (0.933, 1.072) | 0,235 |
| Therizinosauria | 5 | 1-6 | 0,2167 | 0,849 | (0.688, 1.010) | 1,000 |
| Ornithomimosauria | 15 | 1-6 | -0,365 | 1,072 | (0.905, 1.239) | 0.952 |
| Compsognathidae | 7 | 1-6 | -0,106 | 0,913 | (0.591, 1.235) | 0,877 |
| Tyrannosauroidea | 23 | 1-6 | 0,224 | 0,761 | (0.506, 0.963) | 0,909 |
| Tyrann. + Megaraptora | 25 | 2, 4, 6 | 0,249 | 0,761 | (0.542, 0.979) | 0,828 |
| Allo. + Megaraptora | 8 | 1, 3, 5 | 0,497 | 0,695 | (0.161, 1.230) | 1,074 |
| Megalosauroidea | 5 | 1-6 | -0,268 | 0,991 | (0.940, 1.041) | 1,168 |
| Ceratosauria | 9 | 1-6 | -0,003 | 0,861 | (0.544, 1.177) | 0,893 |
| Coelophysoidea | 10 | 1-6 | -0,453 | 1,068 | (0.858, 1.279) | 1,001 |
| Non-Maniraptoriform Tetanurae | 44 | 1-6 | -0,025 | 0,868 | (0.781, 0.954) | 1,350 |
| Non-Maniraptoriform Tetanurae excluding Tyrann. | 21 | 1-6 | -0,113 | 0,917 | (0.843, 0.990) | 0,966 |

**Table S14:**

Pooled linear regressions using the reduced dataset for specific subclades under OU. N is the number of specimens, Intercept, Slope and Slope 95% CI are estimations obtained after pooling 1000 dichotomous time-scaled trees generated for the specified topology or topologies. α is a simple mean of α estimations on the same regressions. Only subclades represented by more than three specimens were included in this table. Top. = Topologies, Tyrann. = Tyrannosauroidea, Allo. = Allosauroidea.

| Clade | N | Top. | Intercept | Slope | Slope 95% CI | α |
| --- | --- | --- | --- | --- | --- | --- |
| Scansoriopterygidae | 3 | 1-6 | -0,060 | 1,077 | (-35.886, 38.040) | 2,457 |
| Troodontidae.+ *Anchiornis* related | 13 | 1-2 | 0,623 | 0,603 | (0.375, 0.830) | 2,010 |
|  |  | 3-4 | 0,622 | 0,603 | (0.375, 0.831) | 1,998 |
| *Anchiornis* related | 5 | 1-6 | -0,146 | 1,038 | (0.759, 1.317) | 1,237 |
| Dromaeosauridae + Troodontidae | 23 | 1-2 | 0,014 | 0,915 | (0.726, 1.105) | 1,998 |
|  |  | 3-4 | 0,014 | 0,916 | (0.726, 1.105) | 2,008 |
|  |  | 5-6 | 0,013 | 0,916 | (0.727, 1.105) | 1,998 |
| Troodontidae | 8 | 1-6 | 0,390 | 0,704 | (0.413, 0.995) | 0,960 |
| Droameosauridae | 15 | 1-4 | 0,195 | 0,847 | (0.611, 1.084) | 1,175 |
|  |  | 5-6 | 0,194 | 0,847 | (0.608, 1.086) | 1,083 |
| Dromaoesauridae excluding *Microraptor* | 14 | 1-4 | 0,165 | 0,861 | (0.606, 1.116) | 1,208 |
|  |  | 5-6 | 0,165 | 0,861 | (0.606, 1.116) | 1,102 |
| Oviraptorosauria | 21 | 1-4 | -0,356 | 1,051 | (0.950, 1.151) | 1,074 |
|  |  | 5-6 | -0,352 | 1,049 | (0.948, 1.151) | 0,998 |
| Therizinosauria | 5 | 1-6 | 0,167 | 0,871 | (0.718, 1.024) | 0,116 |
| Ornithomimosauria | 8 | 1-6 | -0,519 | 1,131 | (1.003, 1.259) | 0,965 |
| Compsognathidae | 5 | 1-6 | 0,038 | 0,835 | (0.545, 1.126) | 0,287 |
| Tyrannosauroidea | 12 | 1-6 | 0,218 | 0,761 | (0.495, 1.028) | 1,479 |
| Tyrann. + Megaraptora | 14 | 2, 4, 6 | 0,272 | 0,749 | (0.474, 1.025) | 1,458 |
| Allo. + Megaraptora | 8 | 1, 3, 5 | 0,497 | 0,695 | (0.160, 1.230) | 1,076 |
| Megalosauroidea | 5 | 1-6 | -0,268 | 0,991 | (0.837, 1.145) | 1,564 |
| Ceratosauria | 7 | 1-6 | -0,027 | 0,869 | (0.432, 1.305) | 0,894 |
| Coelophysoidea | 4 | 1-6 | -0,300 | 1,002 | (0.826, 1.177) | 0,987 |
| Non-Maniraptoriform Tetanurae | 31 | 1-6 | -0,022 | 0,867 | (0.772, 0.961) | 1,383 |
| Non-Maniraptoriform Tetanurae excluding Tyrann. | 19 | 1-6 | -0,110 | 0,915 | (0.840, 0.990) | 1,472 |

**Table S15**

Intrespecific and genus specific allometric trends estimated throgh OLS for subclades represented by 3 or more specimens with the addition of *Archaeopteryx lithographica* for comparison. N indicates number of specimens, Slope 95% CI indicates the 95% confidence interval for the estimated slopes.

| Species | N | Intercept | Slope | Slope 95% CI |
| --- | --- | --- | --- | --- |
| *Archaeopteryx lithographica* | 8 | -0.235 | 1.181 | (1.065, 1.297) |
| *Anchiornis huxleyi* | 7 | 0.181 | 0.883 | (0.478, 1.288) |
| *Microraptor* | 7 | -0.146 | 1.056 | (0.585, 1.528) |
| *Microraptor zahoianus* |  | 0.149 | 0.878 | (0.416, 1.341) |
| *Similicaudipteryx yixianensis* | 3 | -0.132 | 0.956 | (0.659, 1.252) |
| *Caudipteryx* | 5 | 0.421 | 0.658 | (-1.362, 2.678) |
| *Caudipteryx zoui* |  | 4.101 | -1.040 | (-16.340, 1.426) |
| *Ingenia yanshini* | 3 | -0.555 | 1.124 | (0.986, 1.262) |
| *Sinornithomimus dongi* | 5 | -0.776 | 1.237 | (0.913, 1.560) |
| *Ornithomimus edmontonicus* | 3 | 1.932 | 0.203 | (-2.765, 3.172) |
| *Gorgosaurus libratus* | 5 | -1.560 | 1.359 | (0.269, 2.449) |
| *Tyrannosaurus rex* | 7 | 1.132 | 0.457 | (0.065, 0.850) |
| *Tyrannosaurus rex* excluding  BMRP 2002.4.1 | 6 | -2.347 | 1.581 | (-0.228, 3.390) |
| *Coelophysis* | 8 | -1.154 | 1.392 | (0.912, 1.873) |
| *Coelophysis bauri* | 7 | -1.549 | 1.576 | (1.204, 1.948) |

**SUPPLEMENTARY DISCUSSION:**

**POTENTIAL ONTOGENETIC TRENDS OF THEROPOD FORELIMB SIZE**

Table S15 shows the results of regressions for species or genera represented by several individuals, that may reflect ontogenetic trends. In most cases regressions did not present any significant allometric trend, with especially wide confidence intervals for *Similicaudipteryx yixianensis, Gorgosaurus libratus* and *Microraptor* (which includes *M. zhaoianus*, *M. gui* and *M. hanqingi*, due to their poorly resolved relationships). *Ingenia yanshini* and *Sinornithomimus dongi* show narrower confidence intervals that suggest positive allometries. If these are the ontogenetic trends, they would be consistent with the evolutionary allometries found for Oviraptorosauria and Ornithomimosauria, respectively.

*Tyrannosaurus rex* showed a significant negative allometric coeffecient. However, this trend was completely dependent on the inclusion of specimen BMRP 2002.4.1, also known as “*Nanotyrannus*”, whose status as a juvenile *Tyrannosaurus* has been disputed [1]. Upon removing this specimen, no clear allometric trend was found for *Tyrannosaurus*, although only a reduced range of sizes was now represented.

Only in *Archaeopteryx lithographica* and *Coelophysis bauri*, there were significant allometric trends with no controversial specimens. The positive allometric coefficient found for *Archaeopteryx* was lower but consistent with previously published ontogenetic allometries [2,3]. *Coelophysis bauri* is an interesting case because, among the specimens considered, many have been positively identified as juveniles [4]. This supports the lack of negative forelimb allometry in the ontogeny of this species, although a dedicated study evaluating ontogenetic stage and size variation is needed.

There is no potential ontogenetic data for Alvarezsauroidea. Although evolutionary trends may help infer ontogenetic trends, there are only two data points (*Haplocheirus* and *Mononykus*) that allow little examination of evolutionary trends, other than noting a marked reduction of both adult body size and forelimb proportions in *Mononykus*, that is not consistent with an evolutionary trend of negative allometry. It therefore seems unlikely that Alvarezsauroids showed negative allometric growth. If proportionally larger forelimbs were not a trait of juveniles in this clade, this could explain why paedomorphosis (as suggested by their bird-like skulls) may not result in adults with larger forelimbs, unlike other subclades with paedomorphic skulls (Oviraptorosauria, Ornithomimosauria, and Aves). Further information on evolution and ontogeny within the Alvarezsauroidea may confirm mosaic evolution in this clade, likely related to the extreme adaptive specialization of the forelimbs.

References

1. Larson P. The Case for Nanotyrannus. In: Parrish JM, Molnar RE, Currie PJ, Koppelhus EB, editors. Tyrannosaurid Paelobiology. Bloomington, Indiana: Indiana University Press; 2013. p. 14–53.

2. Houck MA, Gauthier JA, Strauss RE. Allometric Scaling in the Earliesst Fossil Bird, Archaeopteryx lithographica. Science. 1990;247:195–8.

3. Bennett CS. Ontogeny and Archaeopteryx. J Vertebr Paleontol. 2008;28:535–42.

4. Colbert EH. The Triassic dinosaur Coelophysis. Museum North Arizona Bull Ser. 1989;57:1–160.

**SUPPLEMENTARY FIGURES**

**Figure S1:**

PGLS regressions under OU for humeral against femoral measurements, (A) regressions for the complete dataset before (dotted line) and after (continuous line) excluding subclades that showed significant differences from the general allometric trend after Phylogenetic ANCOVA testing (Oviraptorosauria, Ornithomimosauria, Tyrannosauroidea, Coelophysoidea and Mononykus), (B) regressions for adult (red) and juvenile (blue) specimens


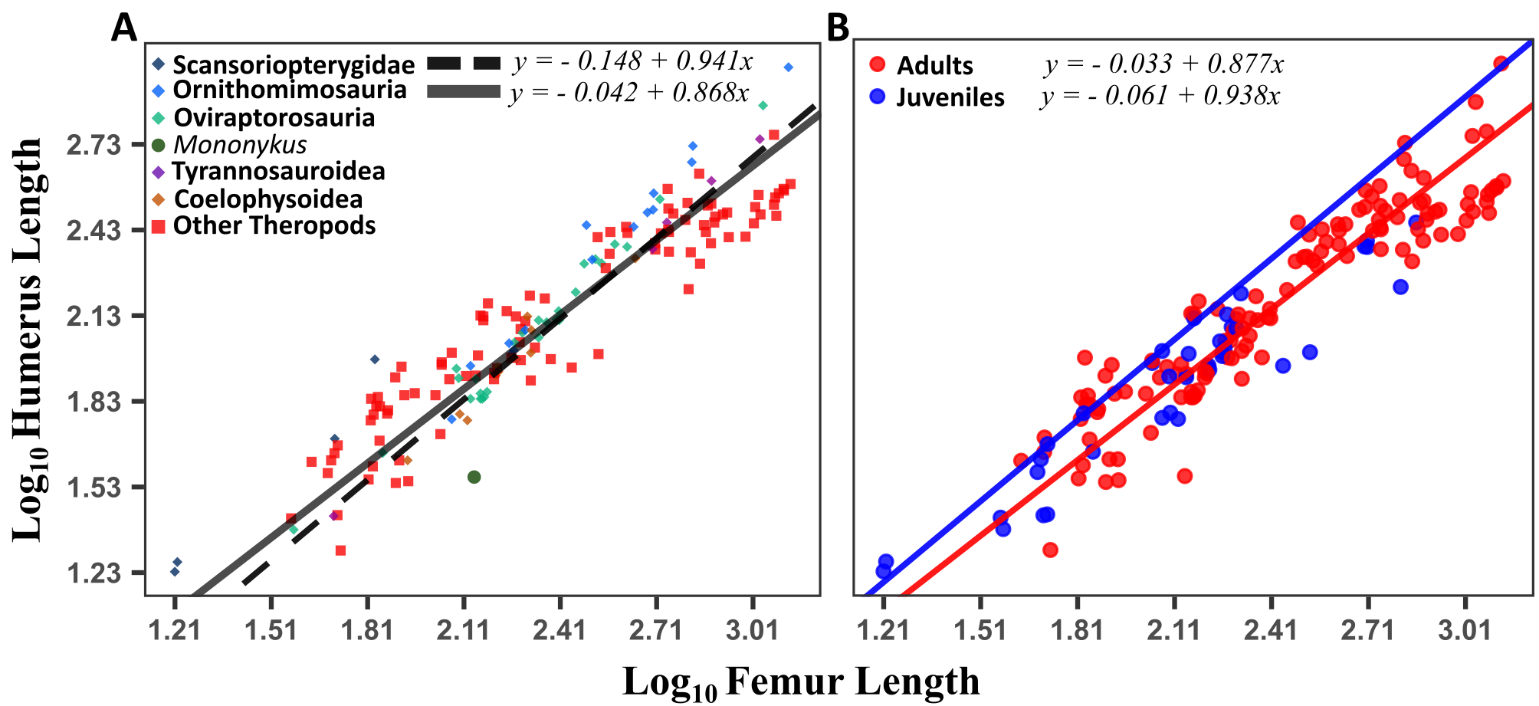


**Figure S2:**

Clade specific allometric coefficients under BM for topologies (A) 1, (B) 3 and (C) 5, bars indicate 95% confidence interval. The subclades that showed significant differences from the general allometric trend of Theropoda through Phylogenetic ANCOVA testing are marked in green (isometry) and red (negative allometry).


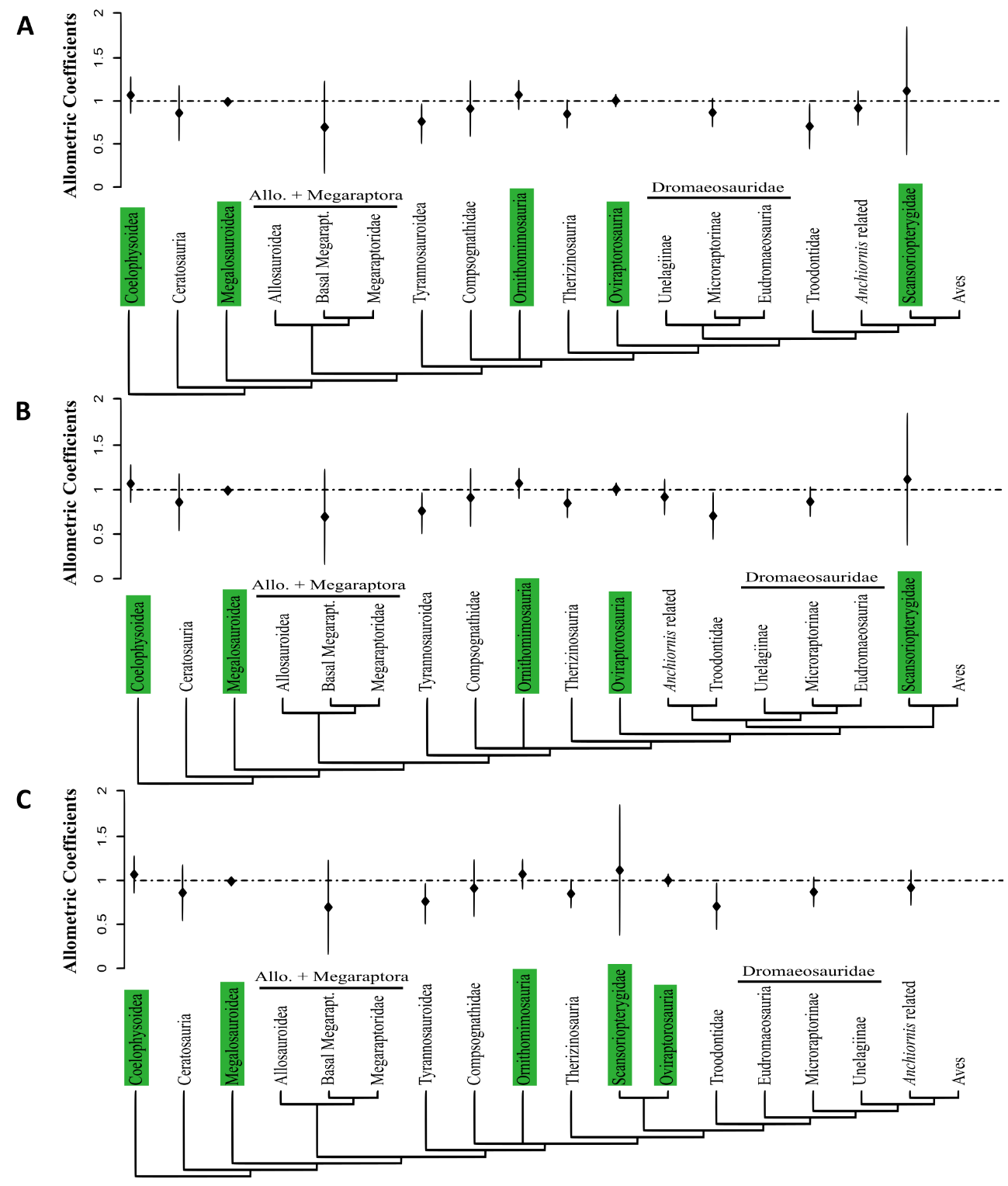


**Figure S3:**

Base topologies used to generate dichotomous time-calibrated trees. Trees based on Senter 2007, Smith et al. 2007, Benson et al. 2010, Zanno 2007, Carrano et al. 2012, Angolín and Novas 2013, Brusatte et al. 2014, Lee et al. 2014, Porfiri et al. 2014, Apesteguía et al.2016, Coria and Currie 2016.


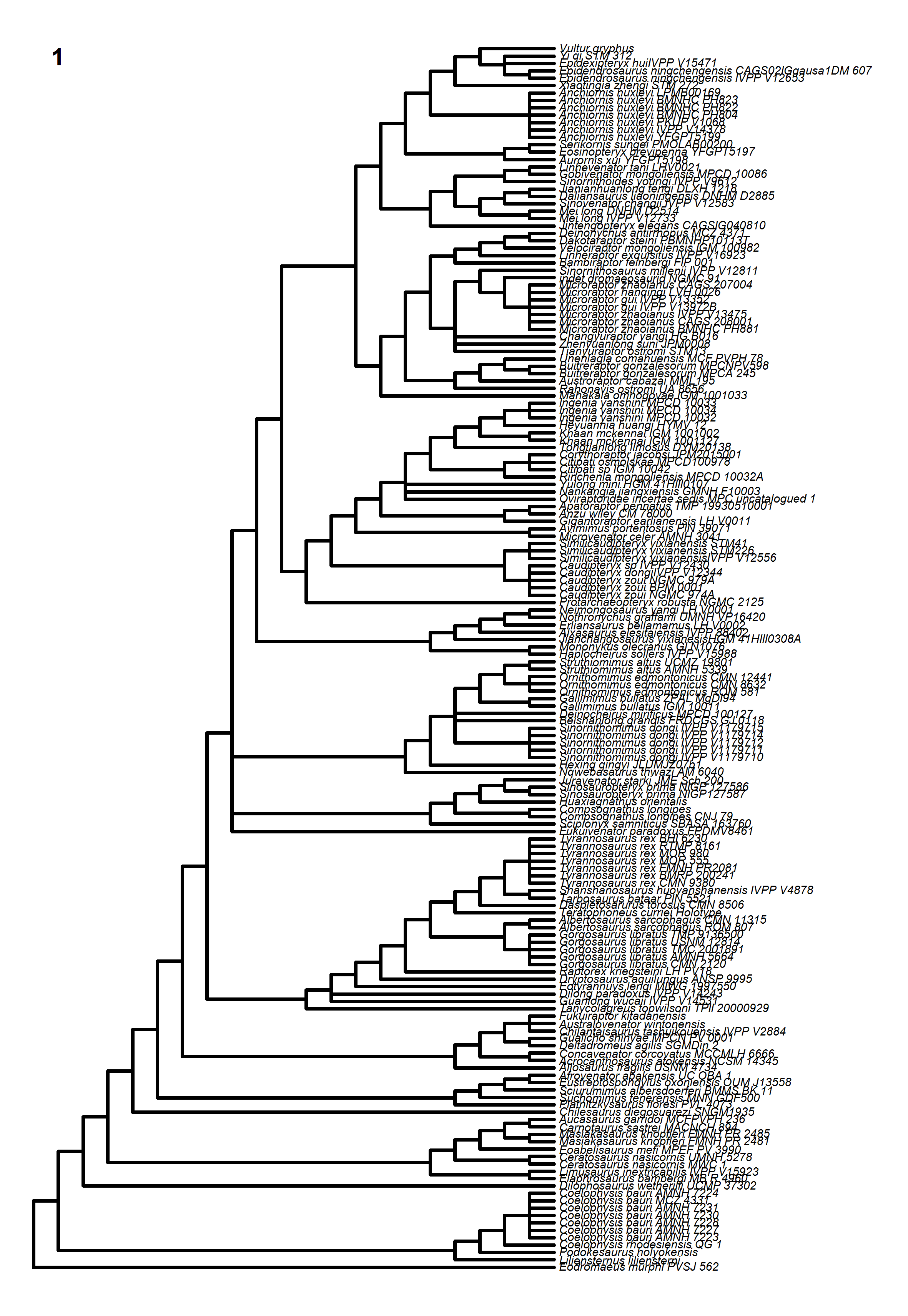


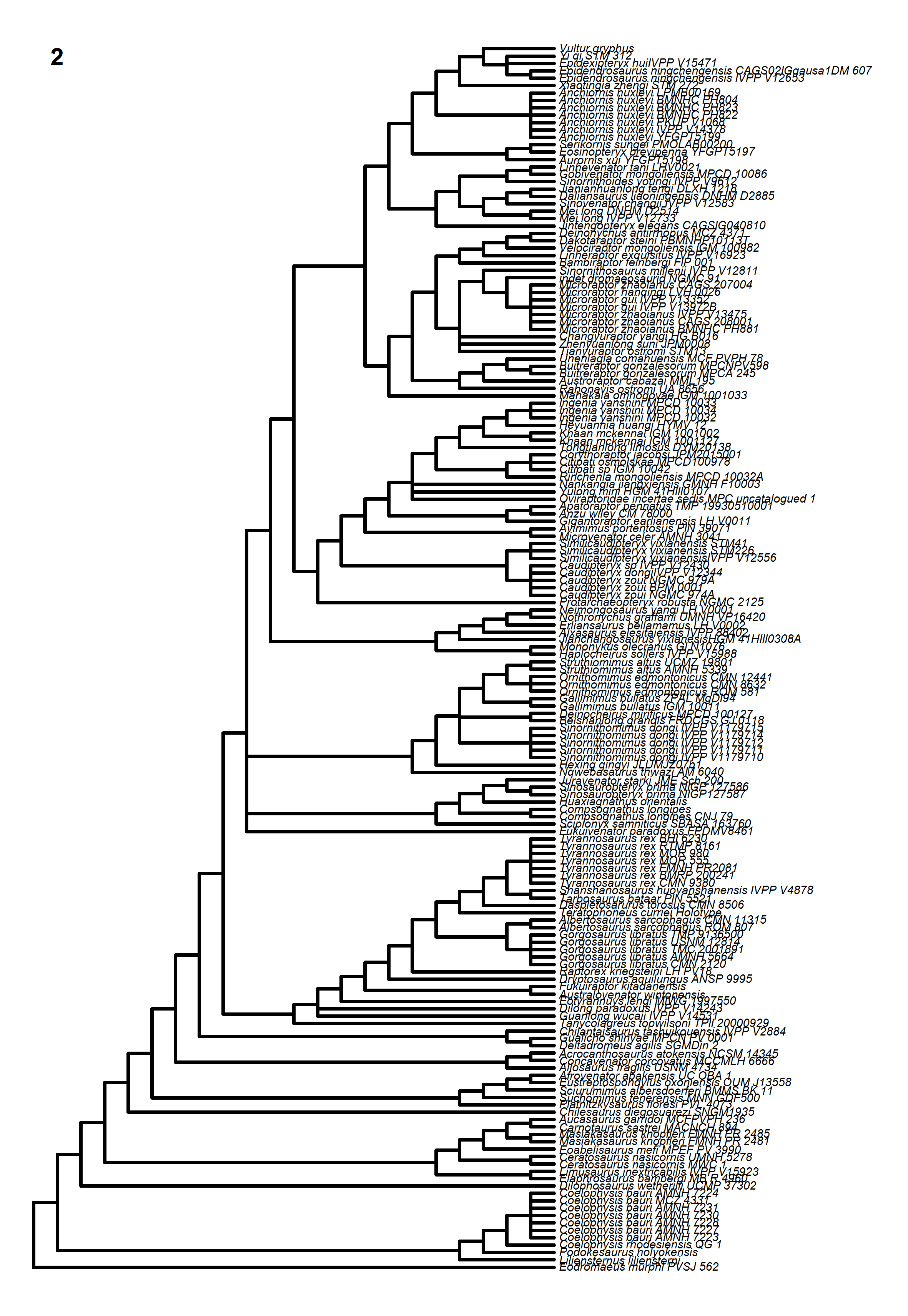


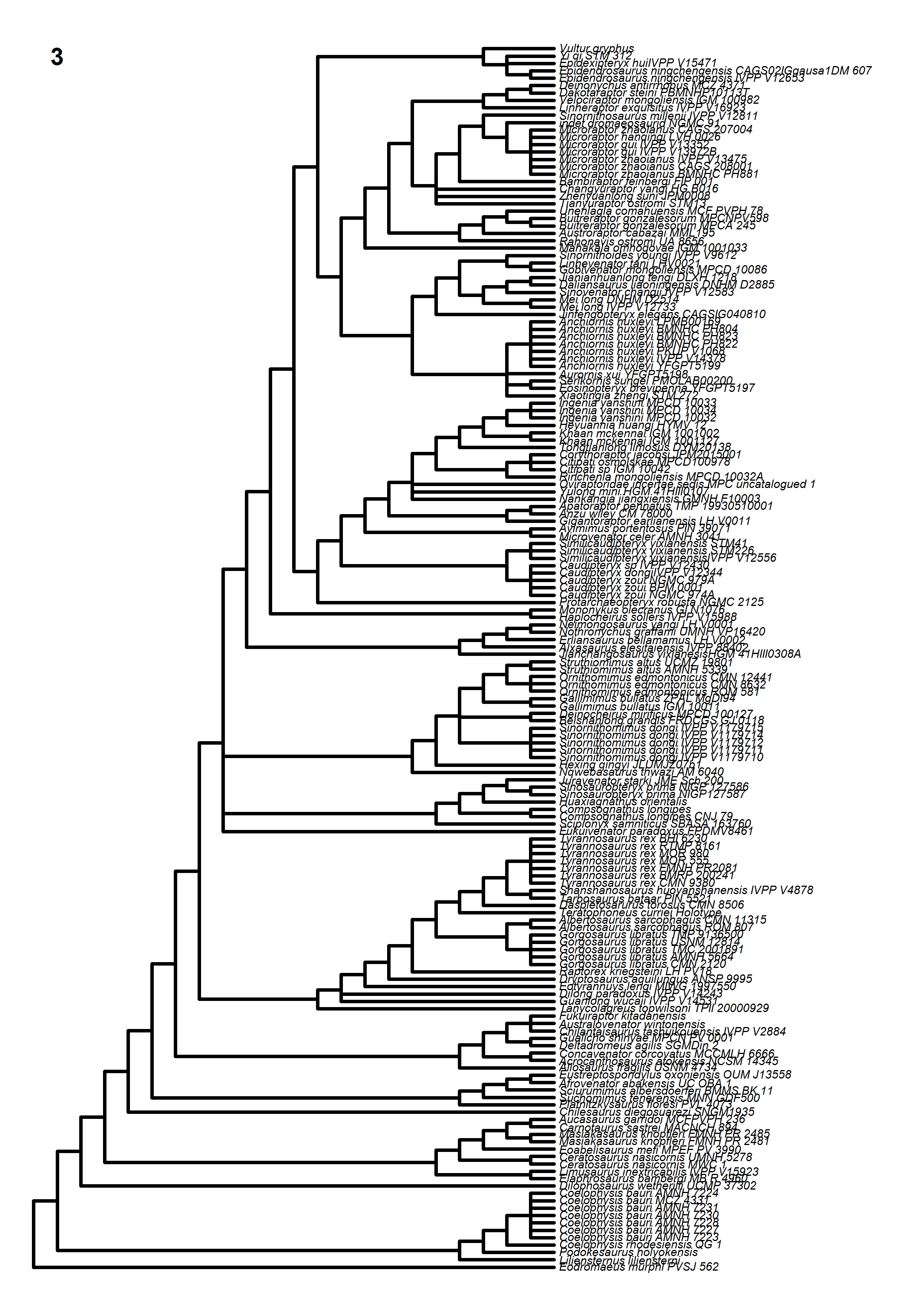


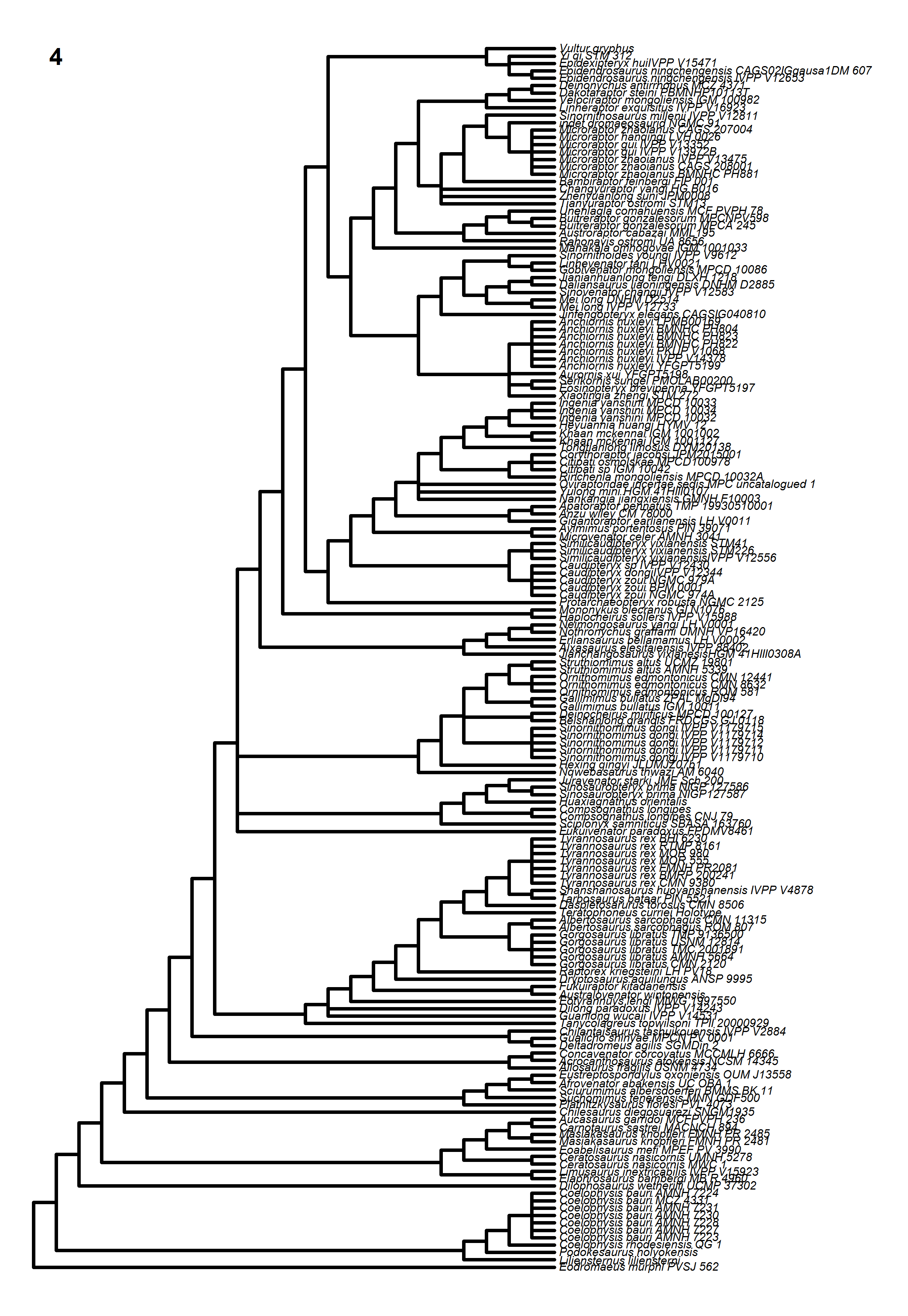


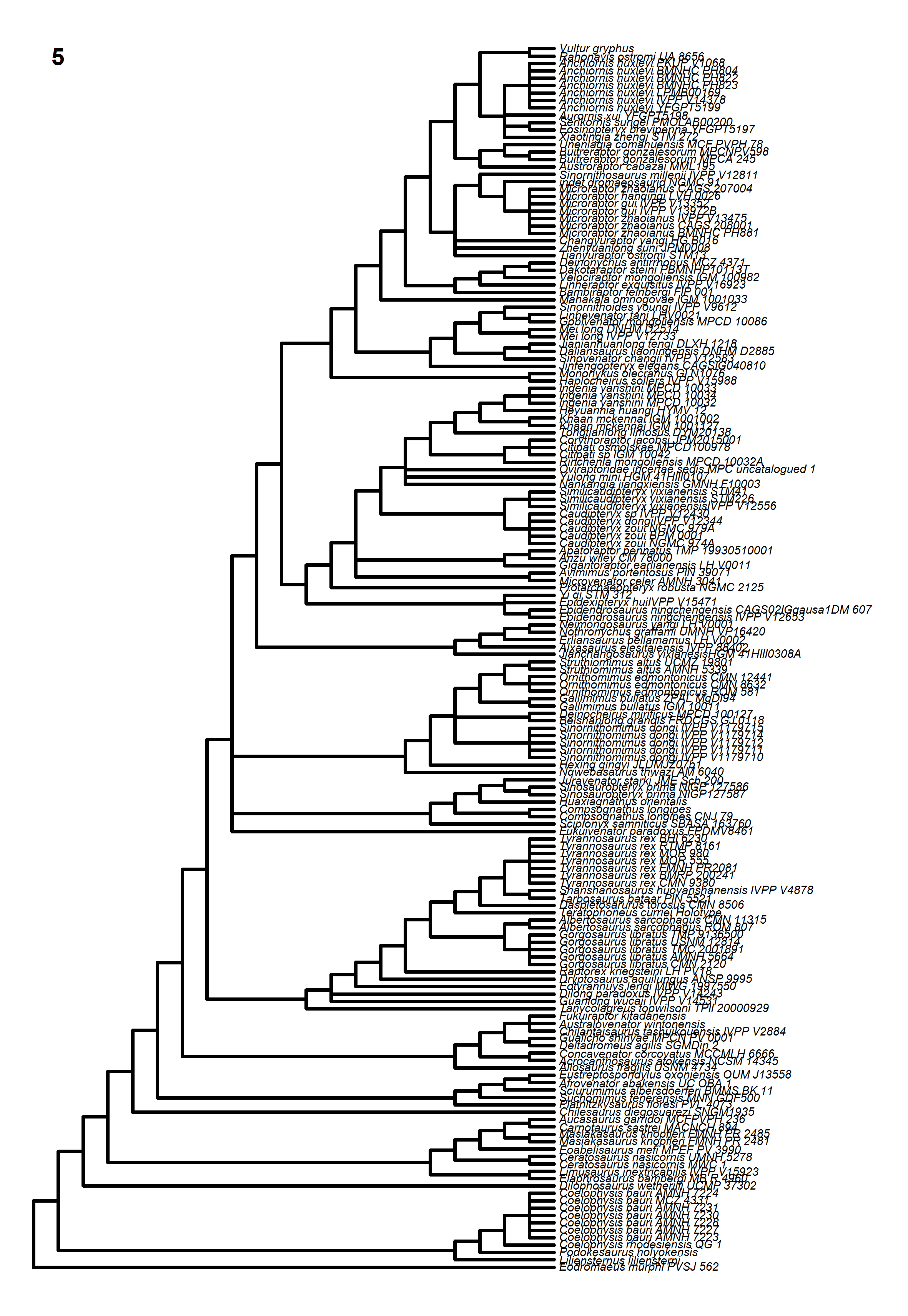


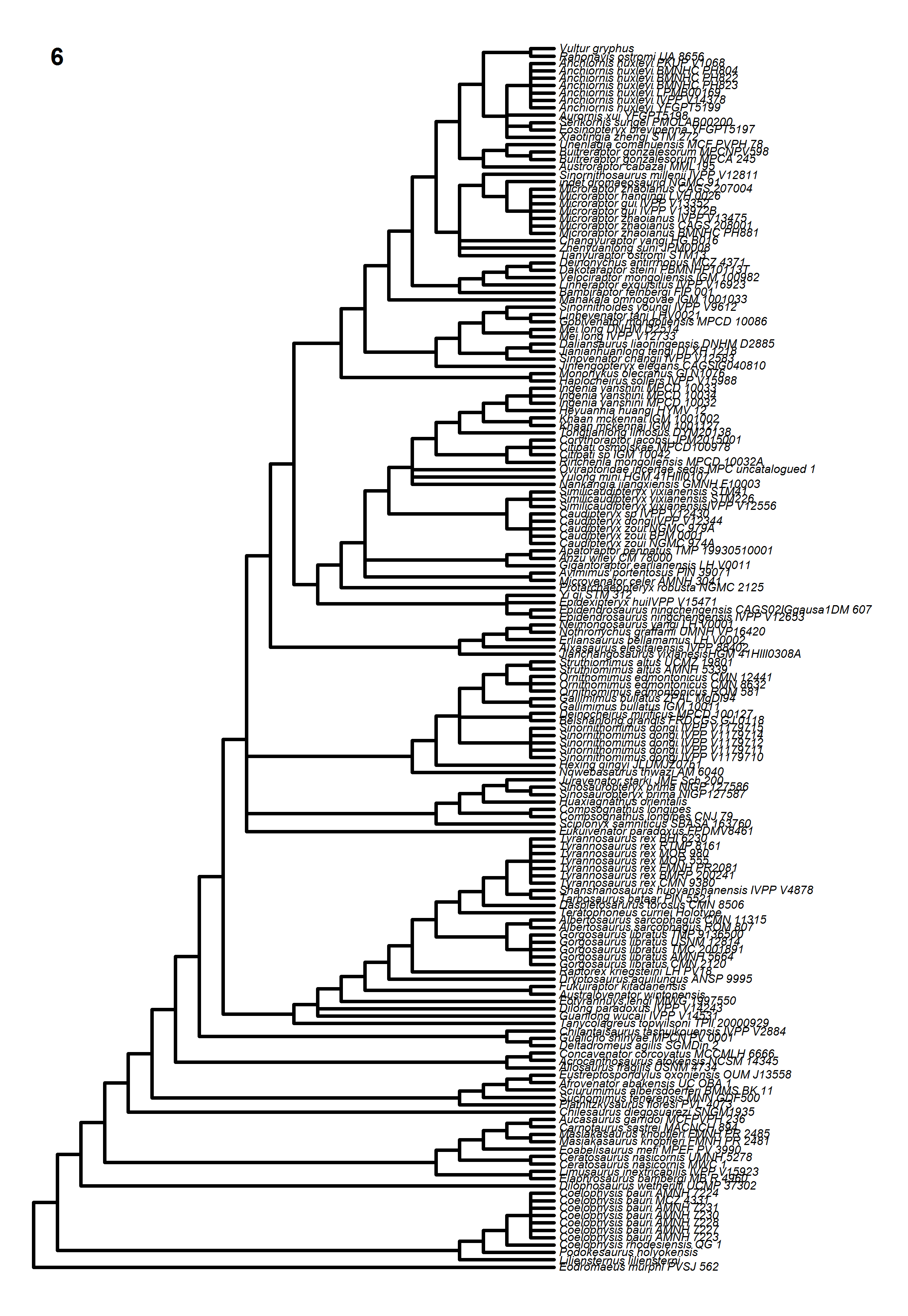

Supplement: Supplementary file 1 — Additional file 1. Supplementary Methods, Tables and Figures [file 12983_2019_342_MOESM1_ESM.docx]
